# Evaluating *Sorghum bicolor* resistance to *Solidago canadensis* invasion under different nitrogen scenarios

**DOI:** 10.3389/fpls.2024.1468816

**Published:** 2024-10-28

**Authors:** Muhammad Anas, Irfan Ullah Khan, Sarah Owdah Alomrani, Mohsin Nawaz, Zhi-Yun Huang, Mohammed Ali Alshehri, Khalid A. Al-Ghanim, Shan-Shan Qi, Jian Li, Zhi-Cong Dai, Shafaqat Ali, Dao-Lin Du

**Affiliations:** ^1^ School of Emergency Management, Jiangsu University, Zhenjiang, China; ^2^ Institute of Environment and Ecology, School of the Environment and Safety Engineering, Jiangsu University, Zhenjiang, China; ^3^ State Key Laboratory of Cotton Biology, Institute of Cotton Research, Chinese Academy of Agricultural Sciences, Anyang, China; ^4^ Department of Biology, College of Science and Arts, Najran University, Najran, Saudi Arabia; ^5^ Department of Biology, Faculty of Science, University of Tabuk, Tabuk, Saudi Arabia; ^6^ Department of Zoology, College of Science, King Saud University, Riyadh, Saudi Arabia; ^7^ School of Agricultural Engineering, Jiangsu University, Zhenjiang, China; ^8^ Jiangsu Collaborative Innovation Center of Technology and Material of Water Treatment, Suzhou University of Science and Technology, Suzhou, Jiangsu, China; ^9^ Department of Environmental Sciences, Government College University, Faisalabad, Pakistan; ^10^ Department of Biological Sciences and Technology, China Medical University, Taichung, Taiwan; ^11^ Jingjiang College, Jiangsu University, Zhenjiang, China

**Keywords:** ecosystem, *S. bicolor*, *S. canadensis*, nitrogen, invasion

## Abstract

Ecosystem exposure to a biological invasion such as plant invasion could contribute to the extinction of native species and loss of productivity and ecosystem balance. *Solidago canadensis* (*S. canadensis*) is a highly invasive species that has formed monocultures in China, Europe, Asia, Australia, and New Zealand. It was designated as a notorious invasive species by the Chinese government. It has adversely affected the agroecosystem’s ability to germinate various plant seeds, including wheat, lettuce, and pepper, which could lead to food insecurity. This study was conducted to control the invasive species *S. canadensis* by utilizing a competitive species, *Sorghum bicolor* (*S. bicolor*) as a cover plant. *Sorghum bicolor* exudes allelochemicals such as sorgoleone from its roots which suppress the photosystem II activity of nearby plants. The synthesis of sorgoleone depends on a supply of nitrogen. The present study involved the cultivation of *S. bicolor* alongside the invasive species *S. canadensis*, with three different invasion levels (high, medium, and low) and three different nitrogen forms (ammonical, nitrate, and combined ammonical and nitrate nitrogen) applied as a modified Hogland solution. *S. bicolor* expressed higher performance over the invasive species under ammonical and combined nitrogen forms under low and medium invasion levels. Furthermore, even at greater levels of invasion, *S. bicolor* was not suppressed by *S. canadensis*. However, the plant height and dry biomass of *S. bicolor* were significantly high across both nitrogen forms. Leaf area, CO_2_ uptake, and photosystem II activity of *S. canadensis* were unable to sustain its growth under the low invasion condition. The plant biomass of *S. canadensis* was suppressed by up to 80% and the relative dominance index of *S. bicolor* was 5.22 over *S. canadensis*. There was a strong correlation between CO_2_ uptake, leaf area, and plant biomass. Principal component analysis showed that the first four components had a total variance of 96.89%, with principal component 1 (PC1) having the highest eigenvalue at 18.65. These promising findings suggested that *S. bicolor*, whose high intensity might be employed to control the invasion process for environmental safety, might be able to recover the barren ground that *S. canadensis* had invaded.

## 1 Introduction

Plant invasion is a major threat to biodiversity, ecosystem balance, and its management. The agroecosystem, which makes up over 40% of the world’s land surface, is extremely vulnerable to invasion (Frost et al., 2019; Batish et al., 2022; Allen et al., 2022). *Solidago canadensis*, native to North America, is the most important and economically significant invasive species in the agroecosystems of Central and Western Europe, Asia, Australia, New Zealand, and China (Anastasiu and Negrean, 2005; Szabó et al., 2020; Qi et al., 2022; Tian et al., 2023). Strong propagation strategies, such as producing a large number of seeds and growing from rhizomes, allow it to dominate an invaded ecosystem (Gazoulis et al., 2022; Khan et al., 2023a). The invasion of *S. canadensis* has put several native species in danger of going extinct (Wang et al., 2021; Gazoulis et al., 2022). It has decreased agricultural productivity, biodiversity, and medicinal plants, and the Chinese government has declared it an exotic invasive species (Wang et al., 2017; Mitryasova and Koszelnic, 2021; Kato-Noguchi, 2023). Thus, sustainable and environmentally friendly management of ecosystems is crucial.

Potential native resources may be sustainable and eco-friendly ways to manage an invaded ecosystem. *Sorghum bicolor* is a strong domesticated plant that can re-sprout and vigorously grow and is mainly grown for its grain and fodder (Venkateswaran et al., 2019). It uptakes nutrients efficiently and grows in diversified soil types such as salt-affected, drought-prone, and water-logged soils (Calone et al., 2020; Chen et al., 2020; Zhang et al., 2023). Crop rotation, root exudates, and water extracts of *S. bicolor* have all been shown to be effective weed control measures in agroecosystems (Zucareli et al., 2019). Because of its effective nutrient intake, allelopathic nature, ability to cover soil, and rapid growth rate during the invasive species’ vegetative growth cycle, it can inhibit *S. canadensis* (Werner et al., 1980; Afzal et al., 2023). It releases hydrophobic and hydrophilic allelochemicals. Sorgoleone is a unique hydrophobic allelochemical, a benzoquinone (2-hydroxy-5-methoxy-1,4-benzoquinone), that is secreted by root hairs, is present in the soil, and exhibits potent phytotoxicity towards a variety of nearby plants (Głąb et al., 2017; Besançon et al., 2020). It is produced throughout the growth period of *S. bicolor*. It inhibits several physiological functions, including the uptake of CO_2_, the transport of electrons in mitochondria, the activity of p-hydroxyphenylpyruvate dioxygenase (HPPD) and H^+^-ATPase in roots, and the uptake of water (Einhellig and Souza, 1992; Weidenhamer, 2005).

Plant-to-plant interaction is highly dependent on plant intensity. Many invasive species have been reported to exert high competition with native species and suppress them. For example, *Erodium cicutarium* invaded a granivore site and dominated all plots (Valone and Weyers, 2019). When invading species become more intense, they release more allelochemicals, which increases competition. *S. canadensis* suppresses *Lactuca sativa* and its suppression depends on the invasion intensity (Yu et al., 2022). Effective plant intensity of *S. bicolor* is important for successful competitiveness between invasive species and *S. bicolor*. It has not been tested against invasive species. However, it was studied to identify the effect of plant intensity on growth, forage, and grain yield and it was concluded that 88,888 plants ha^−1^ was the best intensity to plant at in Ethiopia (Bayu et al., 2005). Yan et al. (2023) reported that a change in plant intensity from 83,000 to 166,000 plants ha^-1^ increased yield by 188 kg ha^-1^ per 10,000 plants.

The root and shoot growth of *S. bicolor* is highly responsive to the form of nitrogen (N). Plants uptake N either in nitrate or ammonical forms (Smil, 1999; Crawford and Glass, 1998). These two forms of N in the soil are interconnected with each other through the ammonification and nitrification processes (Anas et al., 2020). Plants show different adaptations for the uptake of ammonical and nitrate nitrogen forms (Fisher et al., 2010; Du et al., 2020; Zhao et al., 2023). The production of sorgoleone is prolonged by root growth, proton gradient, and H-ATPase under ammonical nitrogen (Di et al., 2018; Afzal et al., 2023). In previous studies, the relation of sorghum root exudates is dependent on the available N form. Afzal et al. (2020) reported that the application of the ammonical form of N improved the production of root exudates. Ammonical nitrogen is biologically nitrified, releasing H^+^ ions and lowering the pH in the rhizosphere soil. Plasma membrane H^+^-ATPase activity increases in the presence of NH_4_^+^ (Zeng et al., 2016). Zhao et al. (2023) reported that sorgoleone had a negative charge which was exuded in the rhizosphere due to the activity of plasma membrane H^+^-ATPase. Sorgoleon production and H^+^-ATPase activity increase under a rhizosphere ammonium concentration of ≤ 0.1 mM (Zeng et al., 2016).

The environment and human health are harmed by the previous ineffective and unsustainable methods of controlling *S. canadensis*, which included burning, eradication, and the use of herbicides (Narwal and Haouala, 2013; Mohd Ghazi et al., 2023). The purpose of this study was to determine the influence of *S. bicolor* on invasive species under different invasion gradients and different nitrogen conditions. We wanted to evaluate to what extent the changes induced by *S. bicolor* are systematic and predictable, and to what extent they are manageable under controlled conditions. In order to investigate how the changes would affect ecosystem services, the productivity of potentially degraded land restored by covering the invasive species and the performance of *S. bicolor* were assessed in an outdoor experiment. It was hypothesized that *S. bicolor* would (a) decrease the growth of invasive *S. canadensis* aboveground and belowground, (b) dominate the invasive plant species by changing their physiological properties, and (c) reduce the dominance of invasive *S. canadensis* compared with sorghum grown under different nitrogen conditions.

## 2 Materials and methods

### 2.1 Experimental site and plant culture

The study was carried out in an outdoor experiment in a greenhouse at the Institute of Environment and Ecology, School of Environmental Science and Safety Engineering, Jiangsu University, Zhenjiang, China from April to September 2023. There is an average of 1,500 mm of precipitation every year in that region, with July having the greatest average of 179 mm and December having the lowest average of 24 mm. The annual maximum and minimum temperatures were recorded as 27.4°C in July and 2.7°C in January (Shen et al., 2023). The riverside is more vulnerable to invasion and sand collected at this site was sieved (2 mm), washed, and sterilized to remove stones, weeds, seeds, and impurities. The pots were filled with 2.5 kg of sand from the Yangtze River, stabilized for 1 week, and wet with double distilled water prior to the transplantation of the seedlings.

The *S. canadensis* seedlings were raised to obtain healthy and similarly sized seedlings. To avoid seed dispersal of the invasive species, *S. canadensis*, seeds of *S. canadensis* were planted in petri plates for germination. The seedlings were then transplanted in plastic trays after 15 days of germination for 2 months to obtain similar two-to-three-leaf seedlings. *S. bicolor* seeds were disinfected with 75% ethanol for 30 s and 10% NaOCl for 10 min and washed thoroughly in double distilled water. Seed disinfection treatment was performed to ensure seed germination and seedlings without seed-born disease. *Sorghum bicolor* seedlings were raised in plastic trays from the disinfected seeds. According to Oswald et al. (2001), the 10-day-old healthy *S. bicolor* seedlings (two to three leaves) and two-to-three-leaf *S. canadensis* seedlings were transplanted into pre-described pots with a 23 cm diameter with plant densities of four *S. canadensis* plants (100% *S. canadensis*: P) per pot, three *S. canadensis* plants and one *S. bicolor* plant (75% *S. canadensis* (H)+25% *S. bicolor* (L) per pot, two *S. canadensis* plants and two *S. bicolor* plants (50% *S. canadensis* (M) + 50% *S. bicolor* (M) per pot, one *S. canadensis* plant and three *S. bicolor* plants (25% *S. canadensis* (L)+75% *S. bicolor* (H) per pot, and four *S. bicolor* plants (100% *S. bicolor*: P) per pot. Following the transplantation of the seedlings into the pots, the pots were kept moist with water for a week before the Hogland solutions were applied. After 1 week after the transplantation of the seedlings, 100 times diluted modified Hogland solutions (**Supplementary Table S1**) with two different forms of available N and their combinations, i.e., 100% NO_3_, 100% NH_4_, and 50% NO_3_+50% NH_4_ concentrations, as well as a control (0% N; CK), were applied at a rate of 100 ml/pot two to three times per week. In total, there were 120 pots with six repeats for each plant density and form of N (**Supplementary Figure S1**).

### 2.2 Plant height, diameter, and root growth traits

The plants in the experiment were harvested at the panicle initiation stage of *S. bicolor*. Before the plants were harvested, the height of five plants was randomly measured using a measuring tape, the number of green leaves was counted, and the plant diameter was measured using a digital Vernier caliber scale (DL91200). The aboveground and belowground parts of the plants were harvested. The dry weight of leaf blades, stems, and roots were noted using a digital weighing balance after the plant tissues were dried at 120°C until constant weight. Root length and root branches were measured according to Khan et al. (2023b). The roots were carefully removed from the pots and washed gently to remove sand media and the root length was measured with measuring tape and root branches were counted.

### 2.3 Gaseous exchange traits

The gas exchange parameters CO_2_ uptake (Pn), transpiration rate (Tr), and stomatal conductance (gs) were measured with a Li-COR 6500 (Lincoln, NE, USA) on a full sunny day between 9:00 am and 11:30 am. The first fully expanded leaf from the top of the plant was selected from both the plant species and the conditions inside the chamber were 1000 µmol m^-2^s^-1^ photon flux density, 400 µmol mol^-1^ carbon dioxide, and a 25°C temperature (Anas et al., 2021b). Water use efficiency (WUE) was measured as a ratio of CO_2_ uptake and transpiration rate.

### 2.4 Chlorophyll fluorescence

The chlorophyll fluorescence of six different plant leaves was measured using a FluorPen (Photon Systems Instruments) for each treatment. Each leaf was clipped after a 20-min dark period. The clip was opened just before recording the electron transfer efficiency (Fm/Fo), potential photochemical activity (Fv/Fo) and maximum photochemical efficiency (Fv/Fm) readings (Davarzani et al., 2023).

### 2.5 Nitrogen content and leaf functions

Leaf N and greenness were measured according to Huang et al. (2022). Briefly, a portable handheld soil plant analysis development (SPAD) meter was used to measure leaf greenness and N content. Three distinct positions were used to assess the nitrogen content and leaf greenness of the first fully expanded leaf of six plants per treatment. Leaf area, perimeter, length, and width were measured using the YMJ-CH intelligent leaf area system (Topu Yunnong Technology, Zhejiang, China) (Cui et al., 2023).

### 2.6 Relative yield and relative dominance index

Relative yield and relative dominance index were measured by Equations 1, 2 respectively (Yau and Hamblin, 1994; Wang et al., 2020; Pan et al., 2023).


(1) RY=Yi/(p×Ymono)

where RY is relative yield, Yi is the yield of species ‘i’ in an interculture, Ymono is the yield of a pure stand, and p is the proportion of species ‘i’.


(2) RDI=Yi/Ytotal

where RDI is the relative dominance index, Yi is the yield of species ‘i’ in an interculture and Ytotal is the sum yield of all species in an interculture.

### 2.7 Statistical analysis

The mean value was calculated for plant height, diameter, root growth traits, gas exchange, chlorophyll fluorescence, nitrogen content, leaf functions, and relative yield and dominance index. The main effects and interactions for the computed mean values of the independent variables were examined using analysis of variance (ANOVA). The calculated mean values were also used as “Trait” values. For every treatment combination, these “Trait” values were converted to the natural log value as follows:


(3) lnR=ln(Trait_x/Trait_y)

where Trait_*x* is the trait value of species *x* and Trait_*y* is the trait value of species *y*. A negative lnR value shows that species *x* is suppressed by the dominance of species *y* (Meng et al., 2015; Huang et al., 2021; Guo et al., 2023).

The lnR values were calculated using Microsoft Excel 2010 and further analysis was performed in RStudio using the “stat” and “ggplot2” packages for ANOVA and the scatter plot, respectively. Pearson’s correlation analysis shows a linear relationship among the variables and principal component analysis (PCA) visualizes the data to identify trends, patterns, or outliers. The “corrplot” and “fviz_pca” functions in R 4.3.2 were used to analyze the correlation and perform PCA, respectively, of the plant height, diameter, root growth traits, gas exchange, chlorophyll fluorescence, nitrogen contents, leaf functions, and relative yield and dominance index lnR values.

## 3 Results

### 3.1 Effect of invasion level of *S. canadensis* and nitrogen form on the phenotype and growth interaction of *S. bicolor*

Plant height, number of leaves, stem diameter, leaf greenness, and N content in the leaf were significantly influenced by the plant species and their interactions with invasion levels and available N forms (**Table 1**; **Supplementary Table S2**). However, the main effects of invasion level, nitrogen form, and interaction of invasion level with available N form were non-significant (**Table 1**; **Supplementary Table S2**).

**Table 1:** Plant height, leaves, diameter, florescence traits, and nitrogen uptake of *S. canadensis* and *S. bicolor* under varied invasion levels and available nitrogen forms for plant species (P), invasion level (I), and nitrogen form (F).

SOV	Df	Plant height	Leaves	Diameter	Fm/Fo	Fv/Fo	Fv/Fm	Nitrogen uptake
**P**	1	629.23**	33.69**	182.27**	10.51**	12.88**	0.08**	57.9**
**I**	3	0ns	0ns	0ns	0ns	0ns	0ns	0ns
**F**	3	0ns	0ns	0ns	0ns	0ns	0ns	0ns
P*I	3	1.22**	0.11**	0.49**	1.67**	7.32**	1.14**	5.63**
P*F	3	6.28**	4.28**	0.72**	0.58**	0.51**	1.09**	1.17**
I*F	9	0ns	0ns	0ns	0ns	0ns	0ns	0ns
P*I*F	9	0.15**	0.12**	0.07ns	0.03**	0.07**	0.02**	0.14**
**Residual**	160	0.02	0.01	0.04	0	0.01	0.01	0.01

Mean square values of plant height, leaves, diameter, Fm/Fo, Fv/Fo, Fv/Fm, and nitrogen uptake; *, ** and ns represented the significance level at p<0.01, p<0.05, and non-significant (p>0.05) respectively. SOV, source of variations; Df, degree of freedom; Fm/Fo, electron transfer efficiency; Fv/Fo, potential photochemical activity; Fv/Fm, maximum photochemical efficiency.

**Figure 1:**
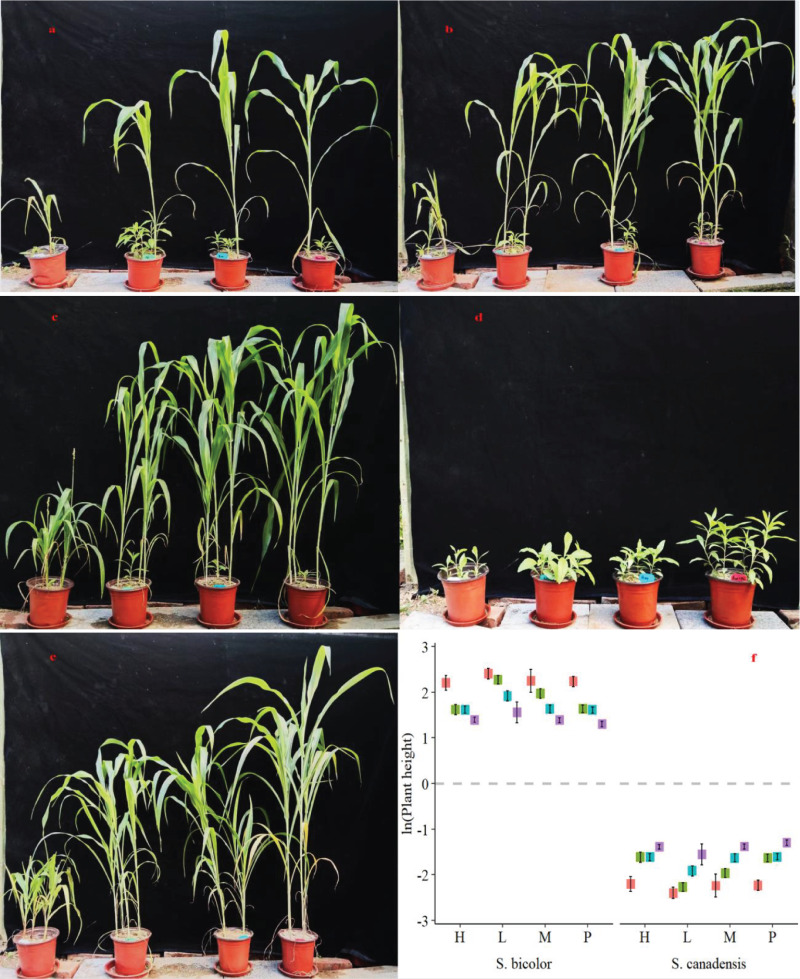
Phenotypic response of *S. canadensis* and *S. bicolor* under varied invasion levels and available nitrogen forms. **(A)** high invasion level; **(B)** medium invasion level; **(C)** low invasion level; **(D)** pure stand of invasive plant species (*S. canadensis*); **(E)** pure stand of domesticated plant (*S. bicolor*); **(F)** relative plant height of both plant species.

The relative plant height of *S. canadensis* (-2.52 to -1.28) was significantly suppressed under the low invasion level of *S. bicolor* for all N forms in relation to the pure stand (**Figure 1F**). The growth of *S. canadensis* was higher under the low population of *S. bicolor* and the NO_3_^-^ N form compared to the medium and high populations of *S. bicolor* (**Figures 1A–C**). However, the highest competitive ability of *S. bicolor* was observed under the combined N form (NH_4_^+^+NO_3_^-^) and a low invasion level (**Figure 1C**). The pure stand of *S. canadensis* showed highest growth under nitrate nitrogen and *S. bicolor* showed under combined nitrogen form (**Figures 1D, E**). Maximum relative plant heights of 2.74 and -1.27 were observed for *S. bicolor* and *S. canadensis*, respectively. Furthermore, across the invasion levels, the height of *S. bicolor* (2.74) was higher under the low invasion level (**Figure 1F**). The respective mean plant heights of *S. bicolor* and *S. canadensis* varied from 61.8 to 15.9 cm and 12.36 to 0.98 cm, respectively (**Supplementary Figure S2A**).

**Figure 2:**
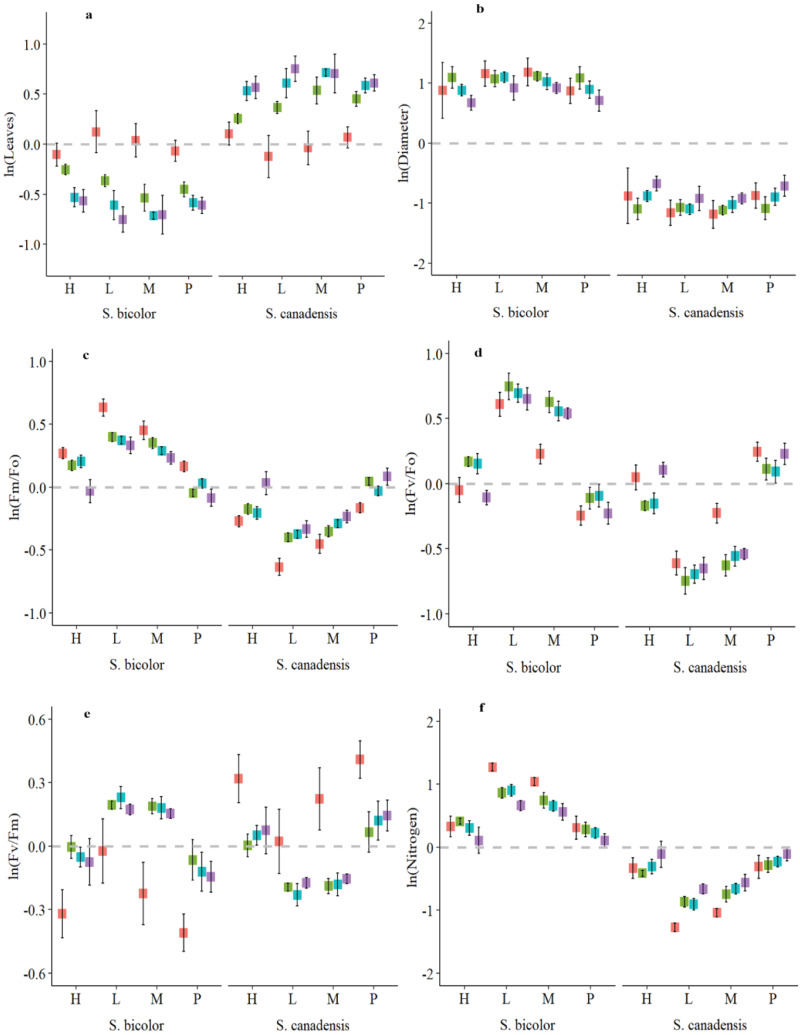
Relative number of leaves **(A)**, stem diameter **(B)**, chlorophyll fluorescence **(C-E)**, and leaf nitrogen **(F)** characteristics of *S. canadensis* and *S. bicolor* under varied invasion levels and available nitrogen forms. Red color, control; green color, ammonical N; blue color, both nitrogen forms; purple color, nitrate N; H, high invasion level; L, low invasion level; M, medium invasion level; P, no invasion.

The relative number of green leaves of *S. canadensis* was higher than *S. bicolor*. The highest relative number of leaves (0.88) of *S. canadensis* was observed against the low invasion level and the NO_3_^-^ N form (**Figure 2A**). The maximum number of green leaves per plant of *S. canadensis* was 16 under the pure culture. This was higher than the number of *S. bicolor* (**Supplementary Figure S2B**). The largest relative stem diameter of *S. canadensis* (-0.39) was noted under the high invasion levels, and the relative stem diameters of the two plant species differed significantly (**Figure 2B**). This was similar to plant height as it was higher under the low invasion levels (**Supplementary Figure S2C**). The leaf greenness (SPAD) values for *S. bicolor* were also higher (52.73) for the low invasion level and both nitrogen forms (**Supplementary Figure S2D**). The relative SPAD value (1.74 to -1.74) was generally influenced across the plant species but no significant difference was observed within a single plant species under all invasion levels and available N forms (**Supplementary Figure S6A**).

For all invasion levels, *S. canadensis* had the largest relative plant leaf N content (0.28 to -0.80) under the nitrate N form; however, at low invasion levels, *S. canadensis* displayed lower relative leaf N content under both N forms (**Figure 2F**). The highest leaf nitrogen content was attained by *S. canadensis* (2.78 mg/g) in the pure culture under both nitrogen forms (**Supplementary Figure S3A**).

### 3.2 Physiological interaction of *S. bicolor* and *S. canadensis* under different invasion and nitrogen conditions

Chlorophyll fluorescence describes photosystem II functionality and Fv/Fm is the ratio of the variable and maximum fluorescence of a dark-adapted leaf. The relative Fv/Fm ratio ranged from 0.18 to -0.28 and from 0.42 to -.26 for low and medium invasion levels for both plant species, respectively (**Figures 2C–E**). It was not significant within the species and was greatly impacted by the application of nitrogen, surpassing the control of both species (**Supplementary Figures S2E, F**).

The gaseous exchange parameters Pn, Tr, and gs were significantly different for the plant species, interactions of plant species with planting intensities, and N form (**Table 2**; **Supplementary Table S2**). The main effect of nitrogen and invasion and the interaction between these two variables were non-significant. However, the significance level of Tr and gs was less in the three-way interaction (**Supplementary Table S2**). Similarly, the fluorescence traits were significant for planting species, interaction of planting species with intensities, interaction of planting species with N forms, and their three-way interaction (**Table 2**; **Table S2**). However, these were unaffected by planting invasion levels and available N forms (**Table 2**, **Supplementary Table S2**).

**Table 2:** Gaseous exchange, water use efficiency, leaf area, and leaf perimeter of *S. canadensis* and *S. bicolor* under varied invasion levels and available nitrogen forms for plant species (P), invasion level (I), and nitrogen form (F).

SOV	Df	Pn	WUE	Leaf area	Leaf perimeter
**P**	1	349.11**	57.36**	1451.12**	851.09**
**I**	3	0ns	0ns	0ns	0ns
**F**	3	0ns	0ns	0ns	0ns
P*I	3	8.06**	1.35**	11.31**	4.32**
P*F	3	2.3**	2.22**	1.09**	0.11**
I*F	9	0ns	0ns	0ns	0ns
P*I*F	9	0.06**	0.04**	0.21**	0.08**
**Residual**	160	0	0.01	0.03	0.02

Mean square values of Pn, WUE, leaf area, and leaf perimeter; *, ** and ns represented the significance level at p<0.01, p<0.05, and non-significant (p>0.05) respectively. SOV, source of variations; Df, degree of freedom; Pn, photosynthetic rate; WUE, water use efficiency.

**Figure 3:**
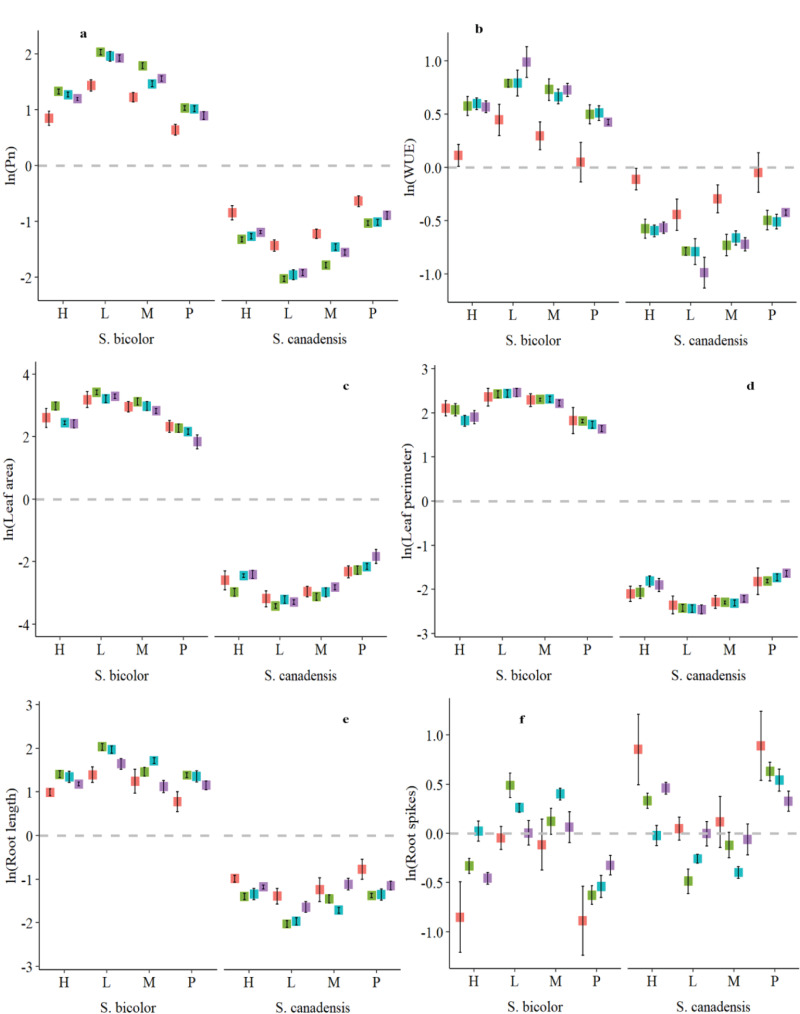
Relative photosynthetic rate **(A)**, water use efficiency **(B)**, leaf area **(C)**, leaf perimeter **(D)**, root dry weight **(E)**, and root spikes **(F)** of *S. canadensis* and *S. bicolor* under varied invasion levels and available nitrogen forms. Red color, control; green color, ammonical N; blue color, both nitrogen forms; purple color, nitrate N; H, high invasion level; L, low invasion level; M, medium invasion level; P, no invasion.

*S. bicolor* had higher relative Pn (2.11) and Tr (1.47) levels compared to *S. canadensis* but gs (-3.35 to -5) was the opposite and Ci was not significant (**Figure 3A**; **Supplementary Figure S6B**). WUE was higher for nitrate N across the plant species and all planting intensities (**Figure 3B**). However, the pure stand for both species and the high invasion level had similar water use efficiency across all the N forms except the no N condition (**Figure 3B**). *S. bicolor* showed non-significant differences for gas exchange parameters under ammonical and combined available nitrogen forms (**Supplementary Figures S3B–F**).

### 3.3 Effect of invasion level of *S. canadensis* and nitrogen form on the leaf area and root growth of *S. bicolor*

Leaf area and its components were also affected only across the plant species and by its interactions with invasion levels and available N forms. (**Table 1**; **Supplementary Table S2**). The maximum relative leaf area for *S. bicolor* (3.55) was observed under the high invasion level and ammonical N form. *S. canadensis* had a maximum relative leaf area (-2.10) under high invasion levels and the nitrate N form (**Figures 3C, D**; **Supplementary Figures S6E, F**). Leaf parameters were influenced by plant species, invasion levels, and nitrogen forms, and the maximum leaf width for *S. bicolor* (26.2 mm) was observed under both nitrogen forms and low invasion levels (**Supplementary Figure S4**).

Root length and root spikes were significantly different for plant species and their interactions with invasion levels and available N forms. However, the largest relative root spikes (1.39) were observed for *S. canadensis* (**Figures 3E, F**). The species-specific root length varied from 64.18 to 4.32 cm for both and it was not statistically significant (**Supplementary Figure S5A**).

### 3.4 Response of biomass and relative indices of *S. bicolor* under different invasion and nitrogen conditions

**Figure 4:**
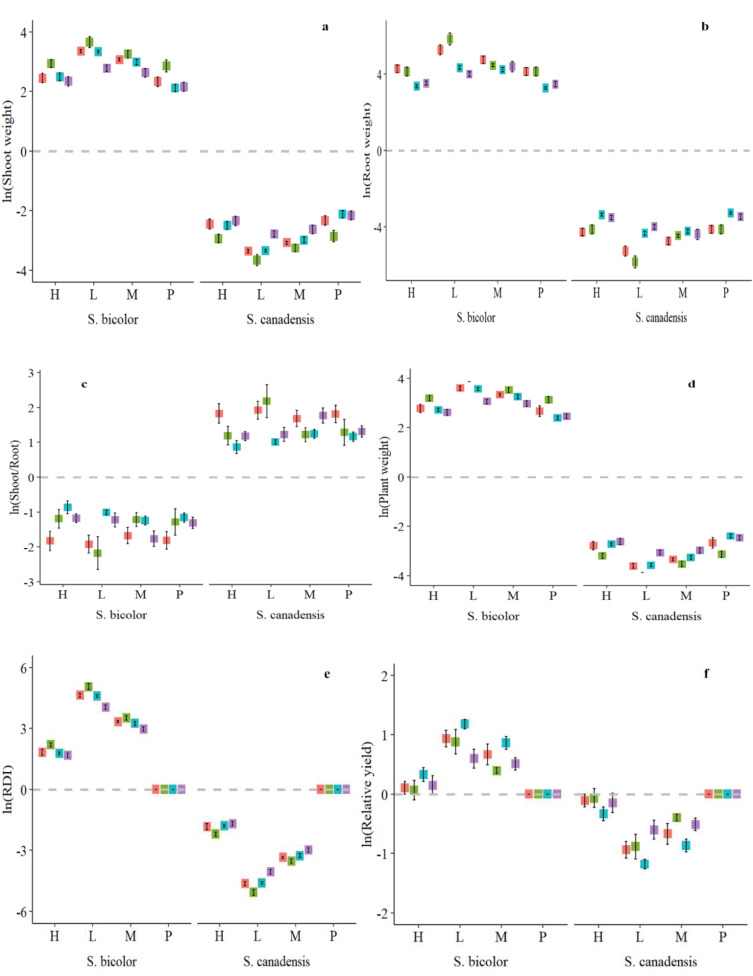
Relative shoot dry weight **(A)**, root dry weight **(B)**, plant dry weight **(C)**, shoot to root ratio **(D)**, relative yield **(E)**, and relative dominance index **(F)** of *S. canadensis* and *S. bicolor* under varied invasion levels and available nitrogen forms. Red color, control; green color, ammonical N; blue color, both nitrogen forms; purple color, nitrate N; H, high invasion level; L, low invasion level; M, medium invasion level; P, no invasion.

The root dry weight was significant against plant invasion levels, N forms, and their interaction (**Table 3**). The shoot/root weight was inversely correlated with the relative dry weight of the plant and, in comparison to ammonical N forms, it was comparable to the control and available N form under both low and high invasion levels (**Figures 4A–C**). The maximum relative plant biomass for *S. bicolor* (4.16) was observed under the high invasion level and ammonical N form and the lowest for *S. canadensis* (-4.16) was observed under the same conditions (**Figure 4D**). Shoot weight, root weight, and total plant dry biomass of *S. bicolor* were similar under the low and medium invasion conditions for the ammonical N form (**Supplementary Figures S5C–F**).

**Table 3:** Root length; root branches; dry biomass of roots, shoots, and total plant; shoot to root ratio; relative dominance index; and relative yield of *S. canadensis* and *S. bicolor* under varied invasion levels and available nitrogen forms for plant species (P), invasion levels (I), and nitrogen forms (F).

SOV	Df	Root length	Root branches	Shoot weight	Root weight	Shoot/root	Total weight	RDI	RY
**P**	1	367.69**	6.03**	1502.33**	0.22ns	392.27**	1825.36**	1138.27**	33.63**
**I**	3	0ns	0ns	0ns	15.74**	0ns	0ns	0ns	0ns
**F**	3	0ns	0ns	0ns	12.39**	0ns	0ns	0ns	0ns
P*I	3	3.39**	7**	8.21**	0.22ns	0.86**	8.13**	185.24**	8.13**
P*F	3	2.75**	2.28**	4.02**	0.22ns	4.49**	3.98**	2.17**	0.78**
I*F	9	0ns	0ns	0ns	1.51**	0ns	0ns	0ns	0ns
P*I*F	9	0.16**	0.41**	0.22**	0.22ns	1.06**	0.19**	0.38**	0.19**
**Residual**	160	0.02	0.03	0.02	0.26	0.06	0.01	0.01	0.01

Mean square values of relative root length, root branches, shoot weight, root weight, total weight, shoot/root, RDI, and RY; *, ** and ns represented the significance level at p<0.01, p<0.05, and non-significant (p>0.05) respectively. SOV, source of variations; Df, degree of freedom; RDI, relative dominance index; RY, relative yield.

The relative yield and RDI for the plant species and their interactions with invasion level and available N forms, and three-way interactions were significantly different (**Table 3**). *S. bicolor* had a higher RDI (5.22) compared to *S. canadensis* even under high invasion levels, and the ammonical and combined N forms (**Figure 4E**). Similarly, *S. bicolor* had a higher relative yield (1.29) under the combined N form, and the relative yield of *S. canadensis* under the low medium and medium invasion levels was lower compared to *S. bicolor* (**Figure 4F**).

### 3.5 Relationship between response variables of *S. bicolor* and *S. canadensis* under different invasion and nitrogen conditions

**Figure 5:**
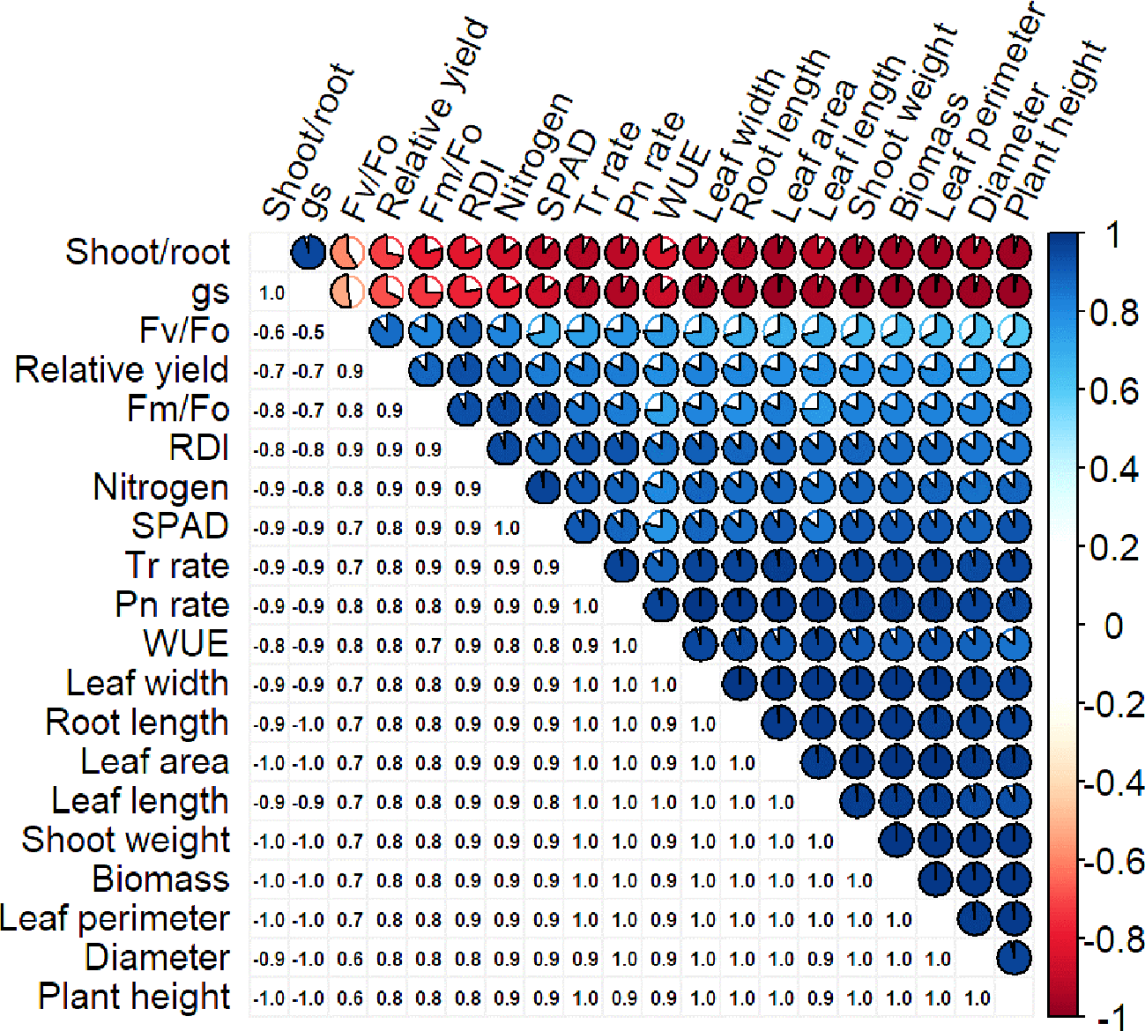
Correlation of different characteristics of *S. canadensis* and *S. bicolor* under varied invasion levels and available nitrogen forms.

Pearson’s correlation coefficient determines the linear relationship between different datasets. A strong Pearson correlation coefficient was observed between Pn rate (0.98), plant height (0.98), and plant diameter (0.98; **Figure 5**). Plant dry biomass also showed a strong correlation with plant height (0.98), diameter (0.98), Pn rate (0.98), Tr rate (0.97), leaf area (0.99), and shoot weight (0.99). However, a weak correlation was observed between biomass and fluorescence parameters (0.67-0.82; **Figure 5**). The leaf N content had a strong correlation with RDI, SPAD, and relative yield, and negative correlations for gs and shoot-to-root ratio were observed against all the parameters (**Figure 5**).

**Figure 6:**
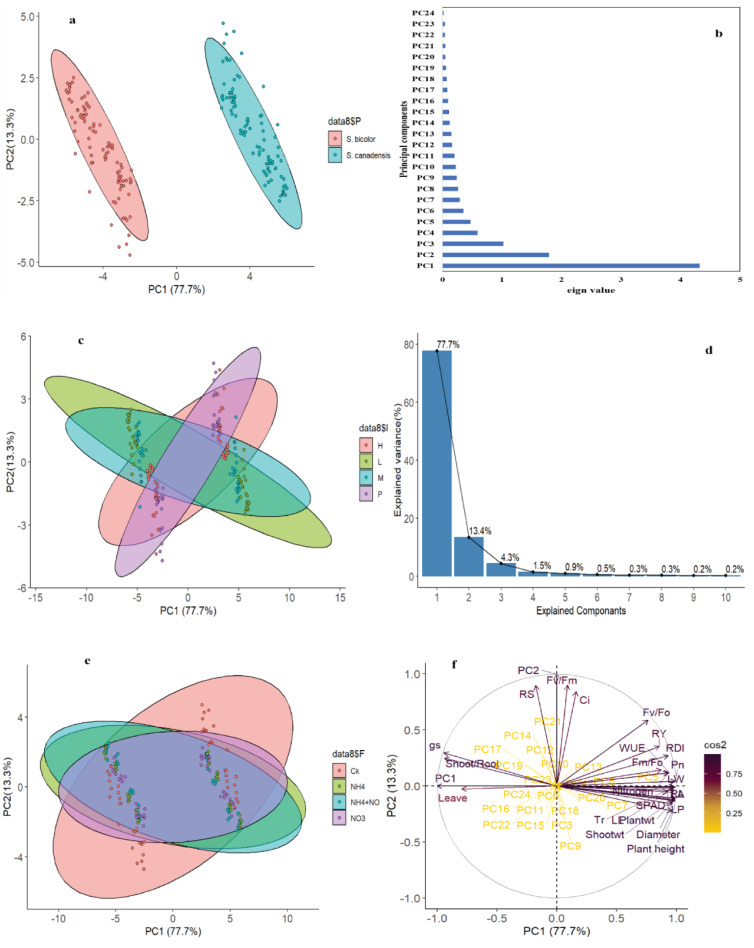
Principal component analysis of *S. canadensis* and *S. bicolor* under varied invasion levels and available nitrogen forms. Biplot of plant species **(A)**, eigenvalue for principal component **(B)**, biplot for invasion levels **(C)**, variance distribution **(D)**, biplot for available nitrogen forms **(E)**, and principal component plot **(F)**.

Principal component analysis separated three treatment factors from 23 components (**Figure 6**). Ellipse grouping showed two, four, and four groups for plant species, plant intensities, and available N forms, respectively (**Figures 6A, C, E**). The cumulative variance for PC1 to PC4 was distributed at 96.89%, and the eigenvalues of PC1, PC2, and PC3 were 18.65, 3.21, and 1.04, respectively (**Figure 6B**). The distribution of the cumulative variance of the top 10 components was displayed using a scree plot (**Figure 6D**). PC1 had the highest cumulative variance, at 77.7%, followed by PC2. The PCA plot showed the relationship of the most relevant principal components (**Figure 6F**). The components with less than a 90° angle were positively correlated with each other and the others were negatively correlated with each other. The PCA plot also showed the contribution of observations to PC1 and PC2 across all the treatment factors respectively (**Figure 6F**).

Except for the plant species, the main impacts of the treatment variables, invasion and N form, were not statistically significant. In the low to high invasion levels, *S. bicolor* exhibited a stronger dominance index over the invasive *S. canadensis* due to its higher plant height, leaf area, root length, and biomass values. Furthermore, the ammonical N form and the combined forms showed a positive relationship with the growth of *S. bicolor* which suggested that it was more responsive to the ammonical N form.

## 4 Discussion

### 4.1 Effect of *S. bicolor* on the growth of *S. canadensis*

*S. bicolor* suppressed the growth of invasive *S. canadensis* by obtaining more height and leaf area and covering the invasive species. Plant dry biomass is the ultimate indicator that describes the successful growth and development of a plant under the provided conditions. Our results showed that *S. bicolor* attained more biomass which supported our first hypothesis that *S. bicolor* would decrease the growth of invasive *S. canadensis* both above and belowground (**Figure 4D**). Allelopathy is an ecological phenomenon in which plants release secondary metabolites that suppress or promote nearby plants. *S. bicolor* is well-established for its allopathy (Farooq et al., 2013). Cheema and Khaliq (2000) reported that *S. bicolor* decreased weed dry biomass by up to 40% in a wheat field. The persistence of *S. bicolor* root exudates also decreased the growth of different weed species (Farooq et al., 2020; Roth et al., 2000). Plant dry biomass is also influenced by plant intensity, for example, the dry biomass of *S. bicolor* was higher in the low plant intensity treatments compared to the high plant intensity treatment but *S. canadensis* under a low intensity had lower plant dry biomass (**Figure 4D**). Furthermore, *S. bicolor* significantly suppressed *S. canadensis* in a combination of high and low *S. bicolor* and *S. canadensis* intensities. This supported our hypothesis that a higher intensity of *S. bicolor* suppresses the growth of *S. canadensis* by changing plant physiology. Similar results were reported by Guo et al. (2023) for *Oenothera biennis*, which could not compete with *Artemisia argyi* in a particular plant population. Previous studies reported that high intensity of *S. bicolor* reduced light interception by weeds (Contreras et al., 2022; Burnside, 1977; Gholami et al., 2013; Besançon et al., 2017a, b). This might be due to low CO_2_ uptake by *S. canadensis* underneath. Similarly, the allelochemicals of *S. bicolor* overcame a weed population by decreasing the production of chlorophyll and the photosynthetic rate (Gonzalez et al., 1997; Jabran and Farooq, 2012; Farooq et al., 2020).

*S. bicolor* is a C4 plant that uptakes CO_2_, water, and nitrogen more efficiently than the C3 *S. canadensis* plant (Zhao et al., 2005; Young and Long, 2000; Anten et al., 1995; Fay et al., 2006). Plant height and diameter have a direct relationship with the application of nitrogen (Anas et al., 2021b). The plant height of *S. bicolor* was also an indicator of its ability to overcome *S. canadensis*, because it covers *S. canadensis* underneath, captures more light and nutrients, and decreases the biomass of *S. canadensis* (**Table 1**; Liu et al., 2022; Ying et al., 2023). Plant diameter was significantly influenced by the planting intensity for both plant species under all N conditions. The results are in line with a previous study that found that a high-intensity plant population resulted in a thin plant stem compared to a low-intensity plant population (Anas et al., 2017).

The roots are responsible for the uptake of nutrients and water as well as interacting with soil bacteria. These secrete the secondary metabolites into the soil and have a direct relationship with the uptake process from the soil (Jiang et al., 2019). Plants produce more roots when grown together with other plants instead of alone and this phenomenon is known as self/non-self root discrimination (Hess and De Kroon, 2007). In this study, root length was found to be significantly different which may be due to the capacity to absorb more nutrients due to close contact with a greater volume of growing media (**Table 3**). Nutrient uptake is dependent on root length instead of root volume. Nutrients in the soil solution are taken up by roots either by mass flow or diffusion (Hodge, 2005). The use of photosynthates is defined by the shoots/roots (Anas et al., 2021a). Compared to *S. canadensis*, the shoots/roots of *S. bicolor* showed a substantial difference, indicating a superior usage of photosynthates (**Figure 4C**). These results were contrary to those of Ren et al. (2019) who found that *S. canadensis* had a greater shoot/root ratio and leaf area allocation with respect to non-invasive plant species. The reason might be that the growth of *S. canadensis* in the early stages was lower when compared to *S. bicolor* (Cox et al., 2018; Kelly et al., 2021). *S. bicolor* had more shoots/roots than *Zea mays* in an interculture with *Panicum millet* and *Zea mays* (Amanullah and Stewart, 2013). *S. bicolor* grew better in drought conditions than *Zea mays* (Danalatos et al., 2009).

### 4.2 Effect of available nitrogen forms on *S. bicolor* overcoming *S. canadensis*

Plant uptake N mainly in two available N forms, either nitrate (NO_3_^-^) or ammonium (NH_4_^+^), and they prefer a particular form according to their adaptation and environment (Sanchez-Zabala et al., 2015). It has been reported that *S. bicolor* prefers the NH_4_^+^ form of N and *S. canadensis* grew better under the NO_3_^-^ form (de Souza Miranda et al., 2016; Miranda et al., 2013; Afzal et al., 2020; Wang et al., 2023). Leaf N content was higher in *S. bicolor* under the combined N and ammonical forms but *S. canadensis* positively responded to the nitrate N form. These results were the same as previous studies that showed that *S. bicolor* is ammonical N-loving and *S. canadensis* is nitrate-loving. *S. bicolor* growth was higher under ammonical N compared to nitrate N due to a low accumulation of H_2_O_2_ and improved K^+^/Na^+^ homeostasis under saline conditions (de Oliveira et al., 2020). Further, Mrid et al. (2016) reported that the application of ammonical N enhanced the growth of *S. bicolor* by improving the activities of glutamine synthetase and aspartate aminotransferase. These enzymes are important parts of the nitrogen metabolic pathway for protein synthesis. However, root growth and exudation of *S. bicolor* increase when ammonical N is present, releasing H^+^ into the rhizosphere and causing the pH to drop (Afzal et al., 2023; Di et al., 2018; Zhao et al., 2023). Root exudates of *S. bicolor* have the potential to retard the growth of nearby plants. The positive responses of *S. bicolor* under ammonical N for protein synthesis and root exudation might enhance its biomass and dominance over the invasive *S. canadensis*. However, another study showed that *S. canadensis* was flexible with regard to available N forms and it tended to take up dominant forms of available N (Guan et al., 2023). Although *S. canadensis* is independent of available N forms, in this study, it gained more biomass under the nitrate N form, similar to Wang et al. (2023) and Afzal et al. (2023). Guan et al. (2023) also used dry biomass as a reference to describe the uptake of a specific N form. Our result supported both arguments as *S. bicolor* acquired the ammonical N form either in the combined treatment or the ammonical form treatment and left less available N for *S. canadensis* due to its dominant growth (**Figure 2F**).

The leaf is the main plant organ that provides the surface for sunlight absorption through chlorophyll pigments and starts photosynthetic metabolism. A high leaf area is the key factor for growth, development, and biomass, and an early development of leaf area is key to gaining more biomass (Liu et al., 2020). The higher leaf area of *S. bicolor* at the early stage might be responsible for its dominance over *S. canadensis* (**Figure 3C**). In this study, we found that leaf greenness was significantly higher in *S. bicolor* under low plant intensity, but higher in *S. canadensis* in the pure stands and at the high invasion level (**Supplementary Figure S6A**). However, it was higher for *S. bicolor* under the combined and ammonical N forms, and *S. canadensis* had higher leaf greenness under the nitrate N form (**Supplementary Figure S6A**). Plants that adopt an N acquisition strategy for the ammonical N form have increased leaf chlorophyll content but excessive application of that form of N might cause chlorosis (Sarasketa et al., 2014; Sanchez-Zabala et al., 2015) because a higher amount of ammonical N degrades the chloroplasts by triggering the ABA signaling pathway (Li et al., 2012). Tomato leaves showed leaf chlorosis through bursting H_2_O_2_ under a medium supply of ammonical N (Liu and von Wirén, 2017).

Plants transform light energy into chemical energy through a process called photosynthesis that occurs in green leaves when they get sunshine. Based on Li et al. (2013), chloroplasts account for 20–30% of the weight of leaves and are the photosynthetic site. One pigment in chloroplasts that absorbs light energy and transforms it into biomass is called leaf chlorophyll (Du et al., 2017). The structural component of proteins, such as chloroplast, is the N. In a previous study, it was reported that the coexistence of N forms had the highest photosynthetic rate which is in line with our results (**Figure 2F**) and followed by nitrate and ammonical N forms (Li et al., 2013). However, nitrate N form was suppressive for photosynthetic rate in this study which might be due to the different carbon fixation pathways of *S. bicolor* (C4) and *S. canadensis* (C3), and C4 plants have higher photosynthetic rates (Smart et al., 2012; Wang et al., 2012; Nawaz et al., 2023).

### 4.3 Dominance and suppressive effect of *S. bicolor* on *S. canadensis*

The relative yield of any plant species shows its position in an ecosystem because it has a direct relationship with successful growth (Leger and Rice, 2003). A change in the behavior of *S. canadensis* due to nearby other plant species describes the invasion level (Conti et al., 2018). The degree of invasion and the evolution of a native species in an invaded ecosystem can be described by an understanding of the adaption mechanism of the invading species (Ren et al., 2022). Relative yield in this study was significantly correlated with plant species, interactions with intensities, N forms, and their combined interaction (**Table 3**). Similarly, *S. bicolor* showed a higher relative yield under the combined N form and low planting intensity. *S. canadensis* did not attain a relative yield equivalent to *S. bicolor* in the intercultures and under all N forms (**Figure 4F**). Thus, choosing the correct plant species is crucial in light of planting intensities and available N sources; these findings are consistent with those of Ren et al. (2022). Agronomic yield is the sum of grains and total dry biomass of plants. It is also calculated on acreage for fodder crops or to calculate an economic return. In this case, ideal plant intensity per acre ensures successful crop growth. Recommended planting intensity utilizes all resources judicially and increases the cost-benefit ratio (Anas et al., 2017). The planting of *S. bicolor* plants in a medium to high intensity may be able to control invasive species according to our testing of plant intensity against various invasion levels in this study. Similarly, de Witt (1960) reported that the yield is dependent on the area available in a mixed culture.

The entry of a non-native species into an ecosystem may change its functions and reshape it for successful invasion and be either partially or fully dominant. The relative dominance of *S. bicolor* based on the plant’s dry biomass was higher than *S. canadensis* for the combined available N forms under low planting intensities (**Figure 4E**). It has been reported that *S. canadensis* may co-exist with non-invasive plant species without changing the species richness, diversity, and composition of the recipient ecosystem (Lai et al., 2015). Usually, invasive plant species change the diversity, species richness, and structure of an ecosystem after entry into that particular ecosystem (Powell et al., 2013; Florens et al., 2017). By developing robust physiological systems both above and below ground, native plant species have the potential to outcompete invasive species. Allelopathy is an ecological strategy used by plants to outcompete neighboring plants by releasing allelochemicals into the air, scattering them on the soil’s surface, and secreting these chemicals from their roots (Farooq et al., 2013; Cheema and Khaliq, 2000; Shan et al., 2023). Plants with greater height and leaf area may cover the vegetation underneath which results in low light penetration and decreases the photosynthetic process (Angadi et al., 2022). Dense populations of plants hinder weed growth in an agroecosystem by stifling their ability to capture resources (Anas et al., 2017). Moreover, plant adaptations for available nitrogen forms may contribute to a native species’ capacity to thrive successfully due to priority (Fisher et al., 2010; Zhao et al., 2023).

## 5 Conclusion

The competitiveness of the domesticated plant *S. bicolor* against the invasive species *S. canadensis* was first documented in this study. It was discovered that *S. bicolor* outcompeted *S. canadensis* because of its greater biomass yield, CO_2_ uptake, leaf area, and plant height. Furthermore, the two plant species exhibited distinct behaviors in response to various N sources. *S. bicolor* performed better under the ammonical N form and combined N form (ammonical+nitrate), and the growth of *S. canadensis* was better under the nitrate N form. *S. canadensis* was suppressed by *S. bicolor* under all interculture combinations and the results were more pronounced at a higher plant intensity of *S. bicolor* under the ammonical N form.

## Data Availability

The original contributions presented in the study are included in the article/**Supplementary Material**. Further inquiries can be directed to the corresponding authors.
