# Supplementary material for: Evaluating Sorghum bicolor resistance to Solidago canadensis invasion under different nitrogen scenarios
Source: Front Plant Sci. 2024 Oct 28;15:1468816. doi: 10.3389/fpls.2024.1468816 (PMC11555567; doi:10.3389/fpls.2024.1468816)
Supplement: Supplementary file 1 [file DataSheet1.docx]

**Supplementary Figures**

**Figure 1S:** Schematic diagram of experiment of *Solidago canadensis* and *Sorghum bicolor* under varied invasion levels and available nitrogen forms, and Ck represent the no nitrogen.


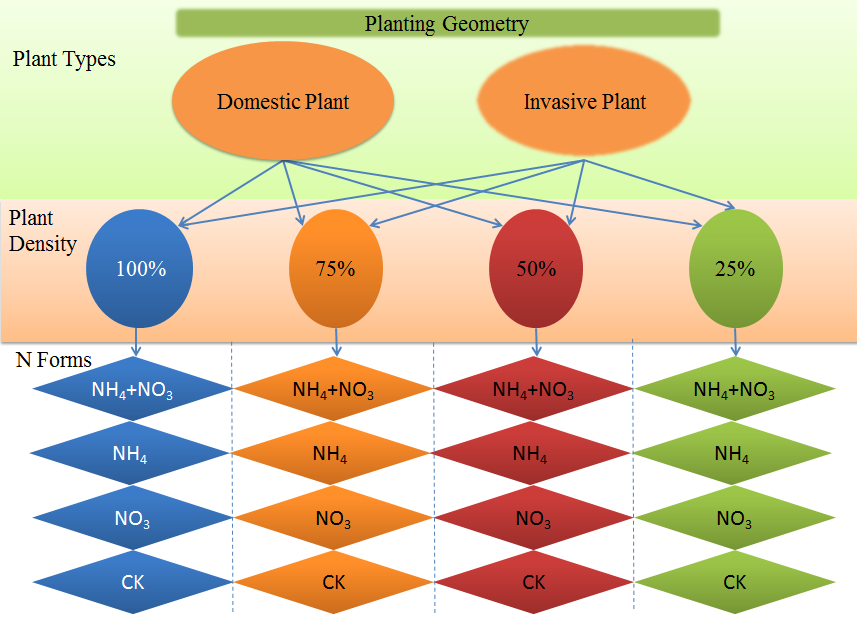


**Figure 2S:** Plant height (a) Number of leaves (b), stem diameter (c), leaf greenness and chlorophyll fluorescence (f) characteristics of *Solidago canadensis* and *Sorghum bicolor* under varied invasion levels and available nitrogen forms. green colour: no nitrogen; yellow colour: ammonical N; purple colour: both nitrogen form; red colour: nitrate N; H: high invasion level; L: low invasion level; M: medium invasion level; P: no invasion.


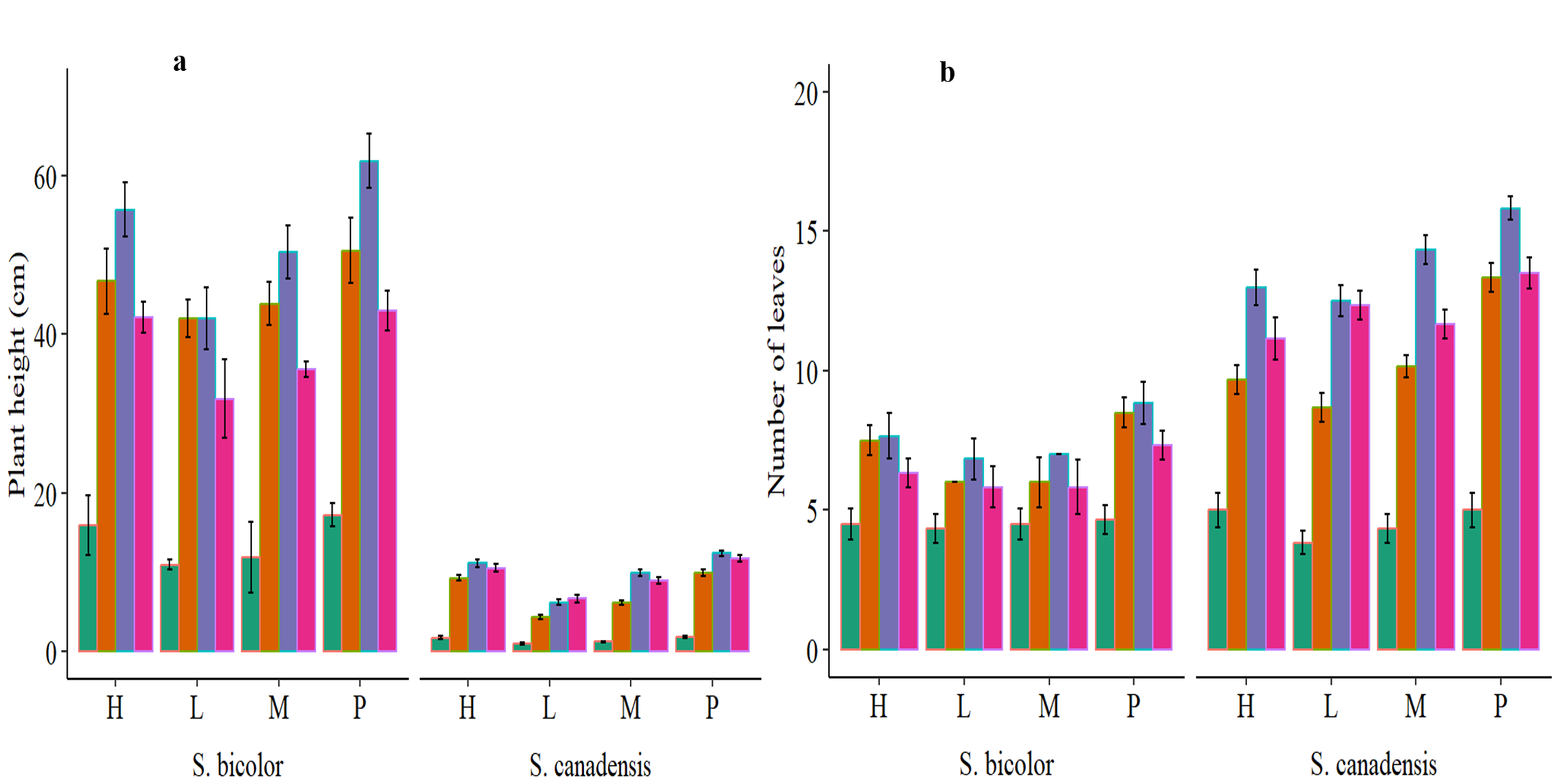

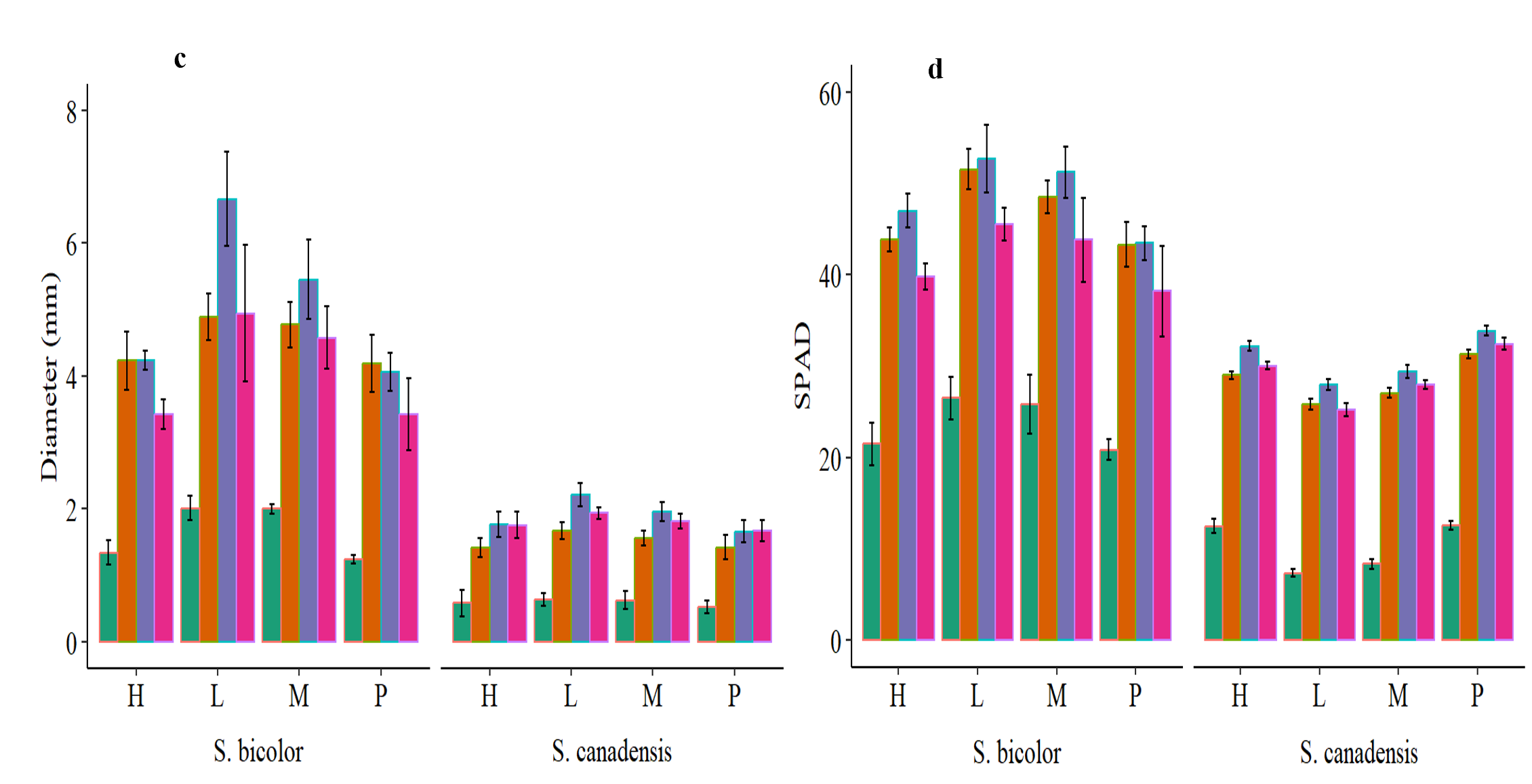

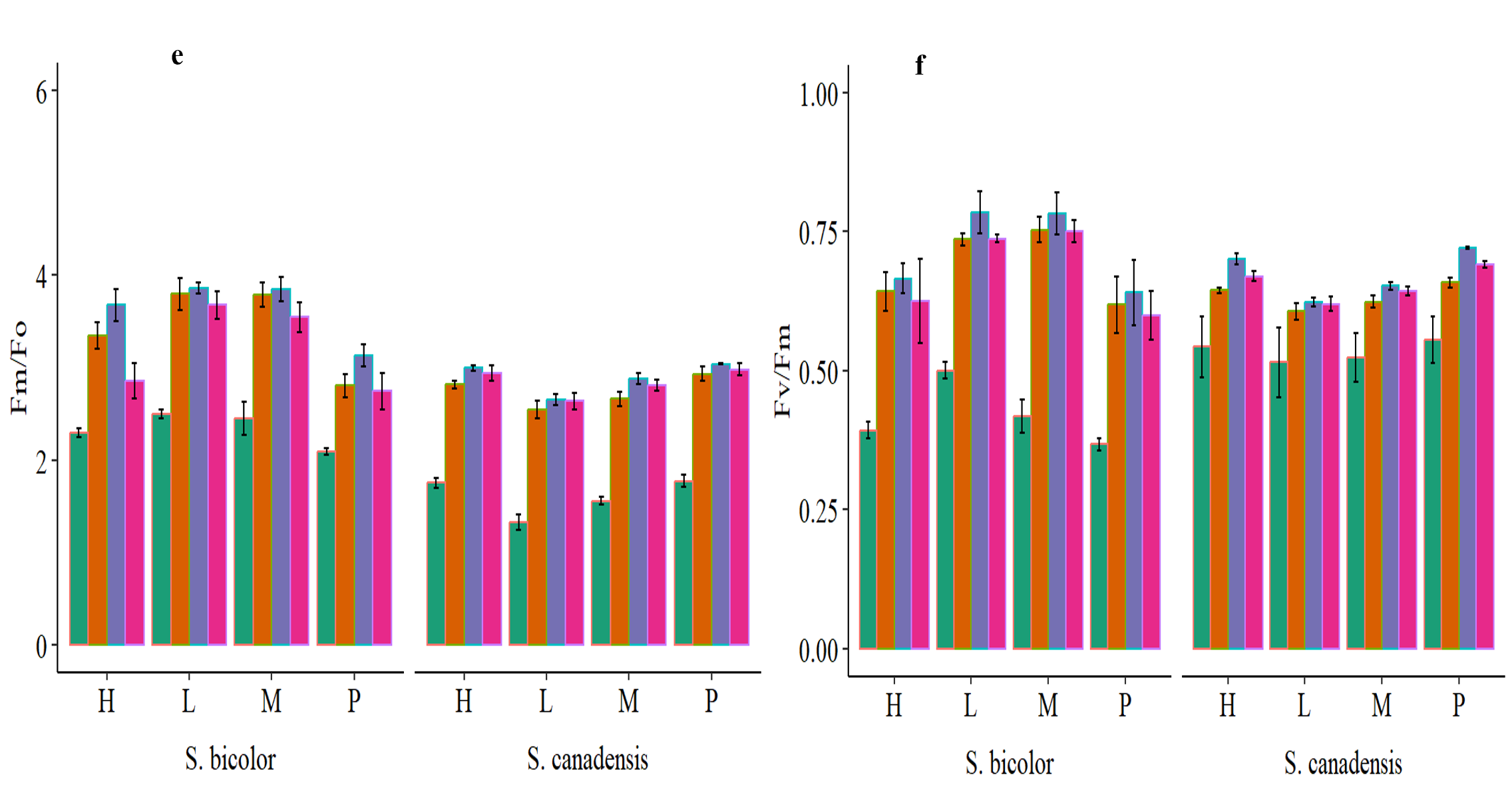


**Figure 3S:** Leaf nitrogen content (a), net photosynthetic rate (b), transpiration rate (c), stomatal conductance (d), intracellular CO_2_ (e) and water use efficiency (f) of *Solidago canadensis* and *Sorghum bicolor* under varied invasion levels and available nitrogen forms. green colour: no nitrogen; yellow colour: ammonical N; purple colour: both nitrogen form; red colour: nitrate N; H: high invasion level; L: low invasion level; M: medium invasion level; P: no invasion.


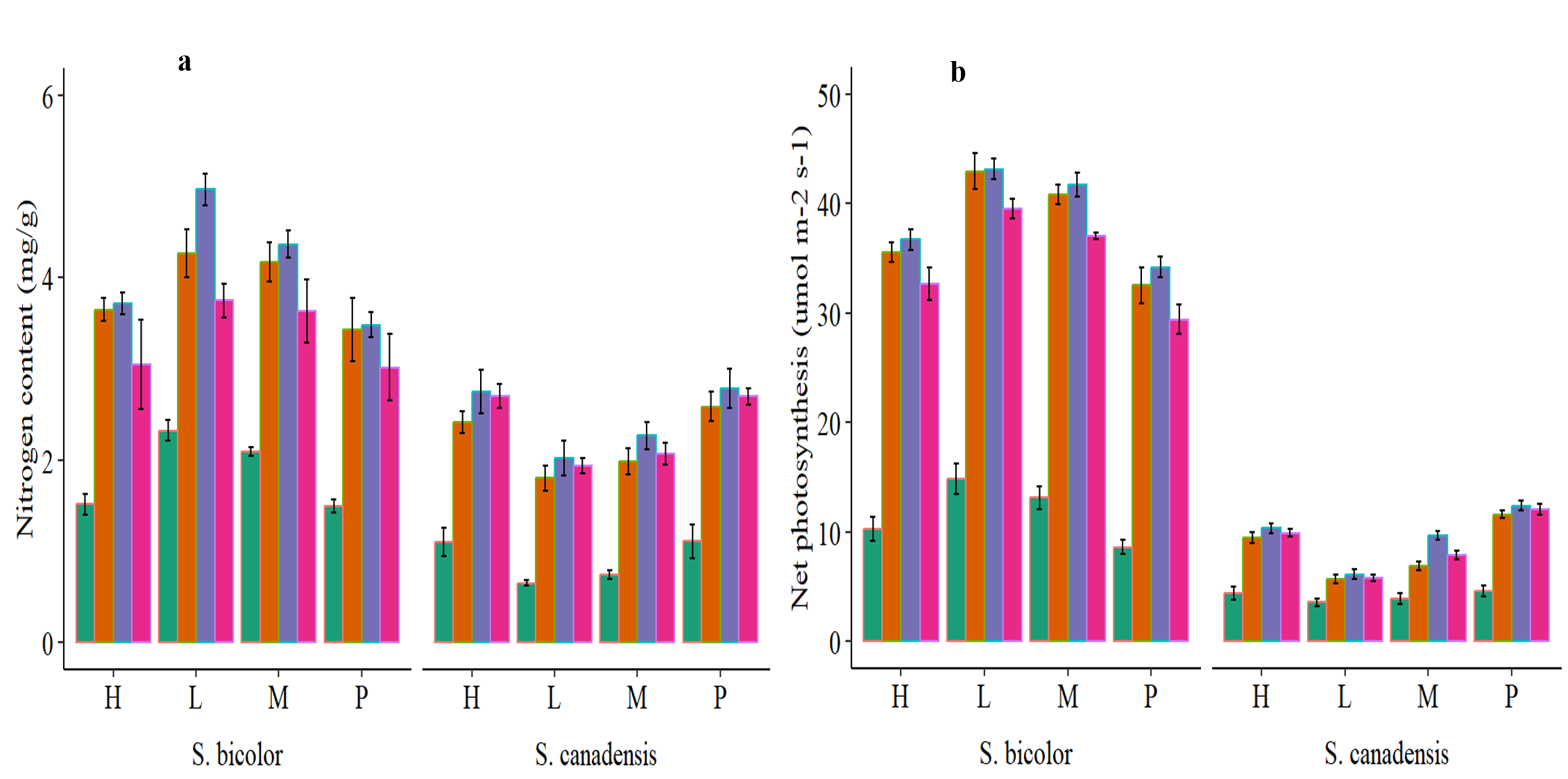

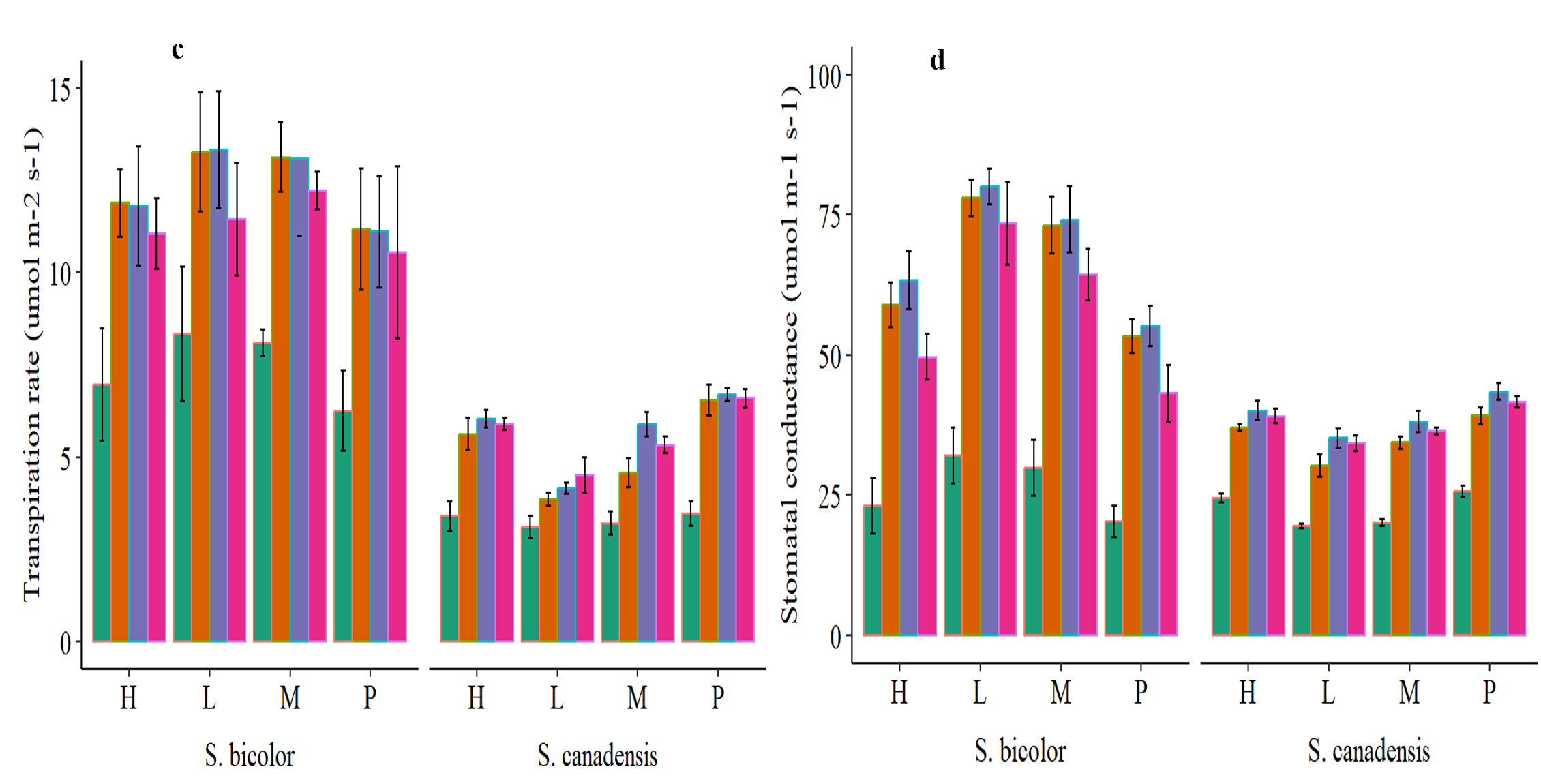

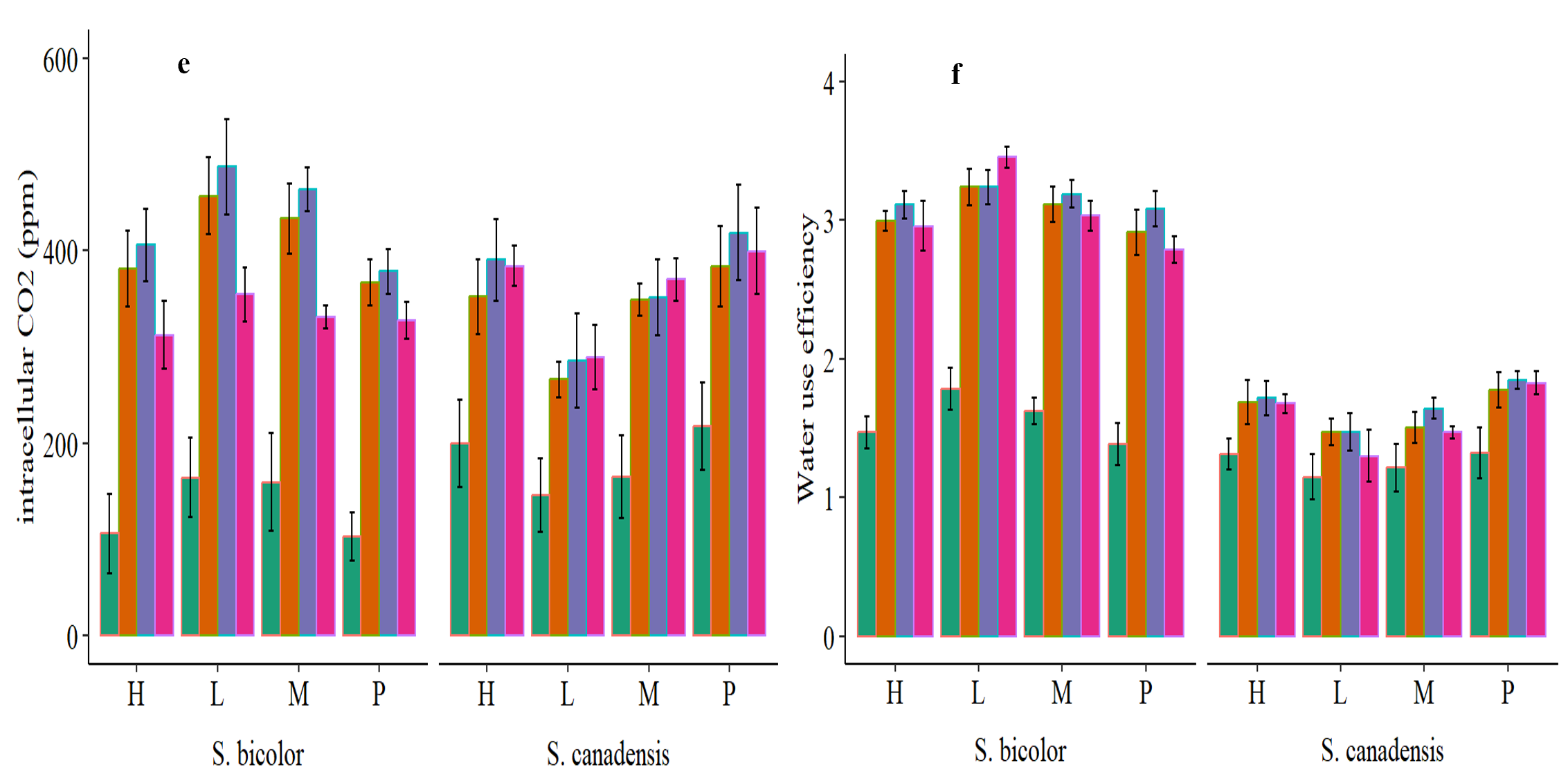


**Figure 4S:** Leaf area (a), leaf perimeter (b), leaf length (c) and leaf width (d) of *Solidago canadensis* and *Sorghum bicolor* under varied invasion levels and available nitrogen forms. green colour: no nitrogen; yellow colour: ammonical N; purple colour: both nitrogen form; red colour: nitrate N; H: high invasion level; L: low invasion level; M: medium invasion level; P: no invasion.


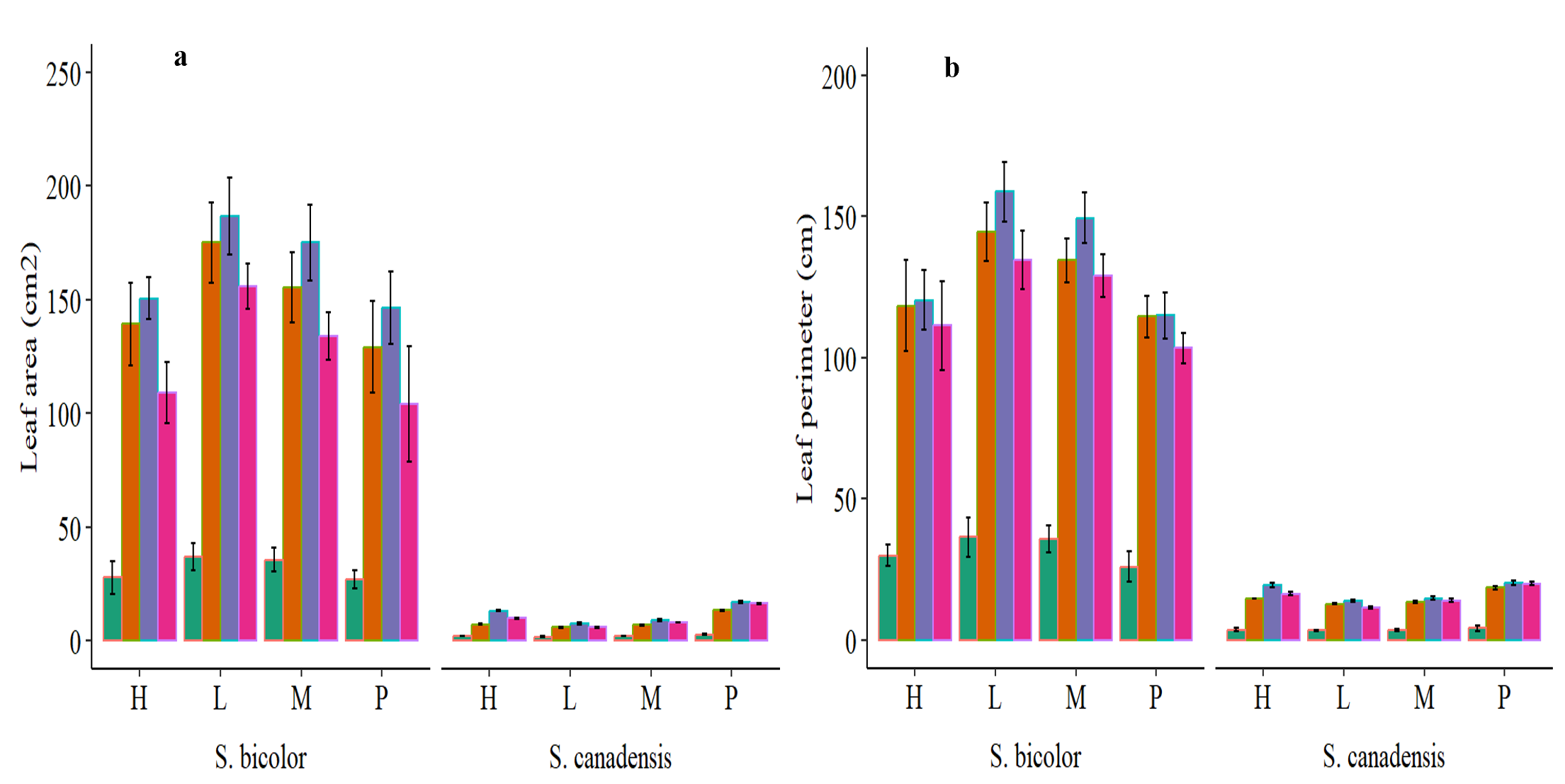

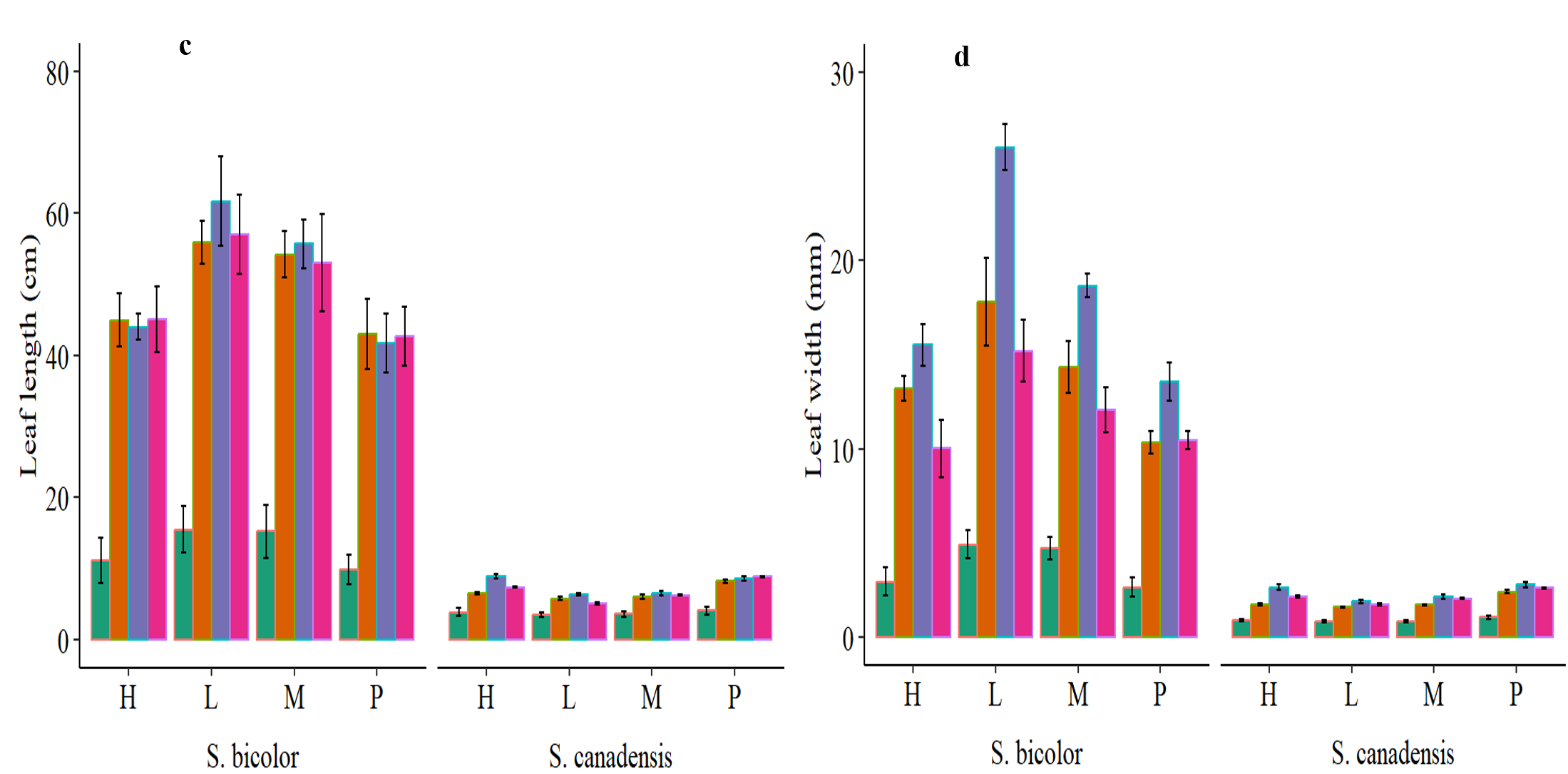


**Figure 5S:** Root length (a), number of root spikes (b) shoot dry weight (c), root dry weight (d), shoot to root ratio (e) and plant dry weight (f) of *Solidago canadensis* and *Sorghum bicolor* under varied invasion levels and available nitrogen forms. green colour: no nitrogen; yellow colour: ammonical N; purple colour: both nitrogen form; red colour: nitrate N; H: high invasion level; L: low invasion level; M: medium invasion level; P: no invasion.


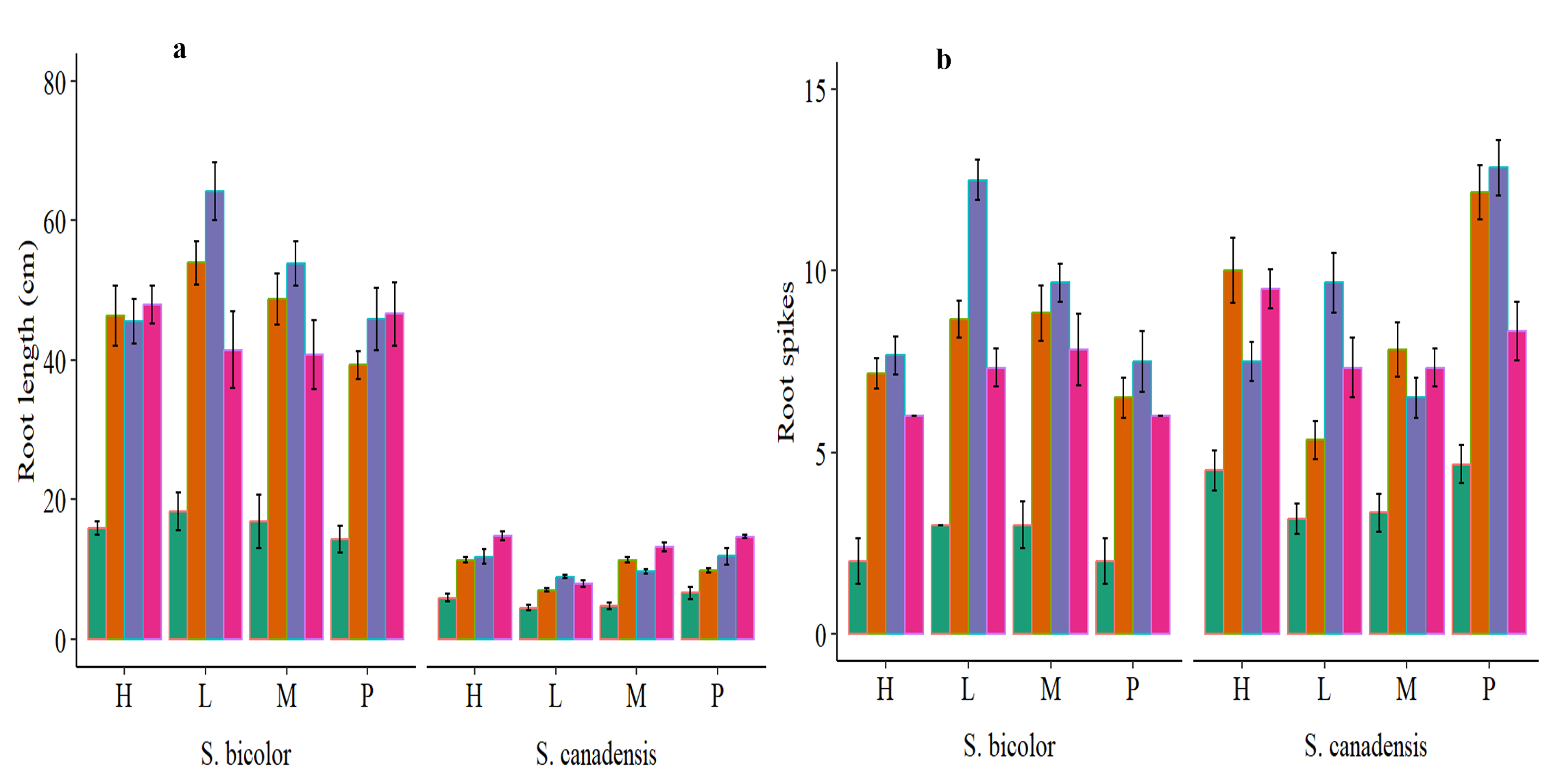

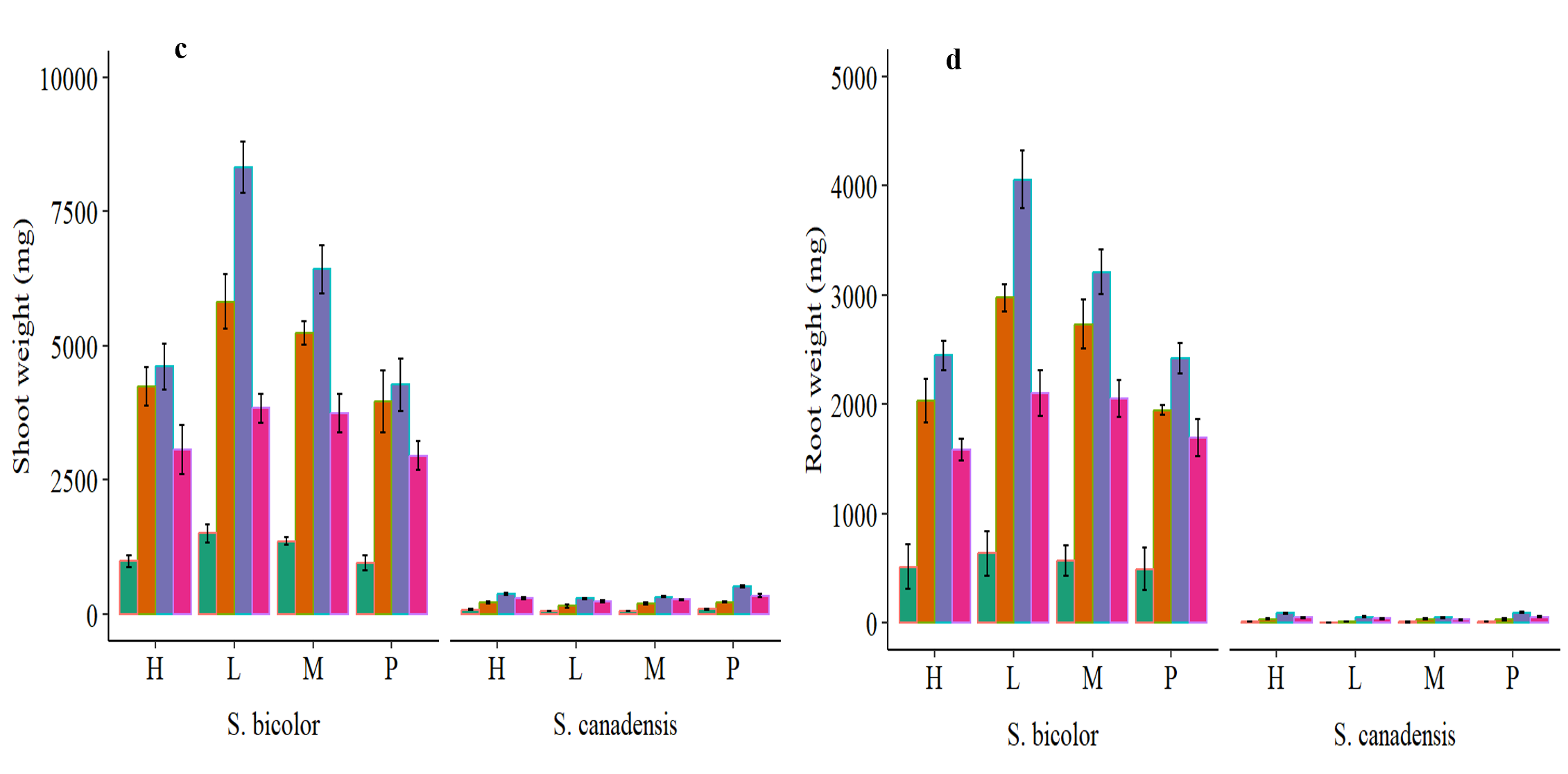

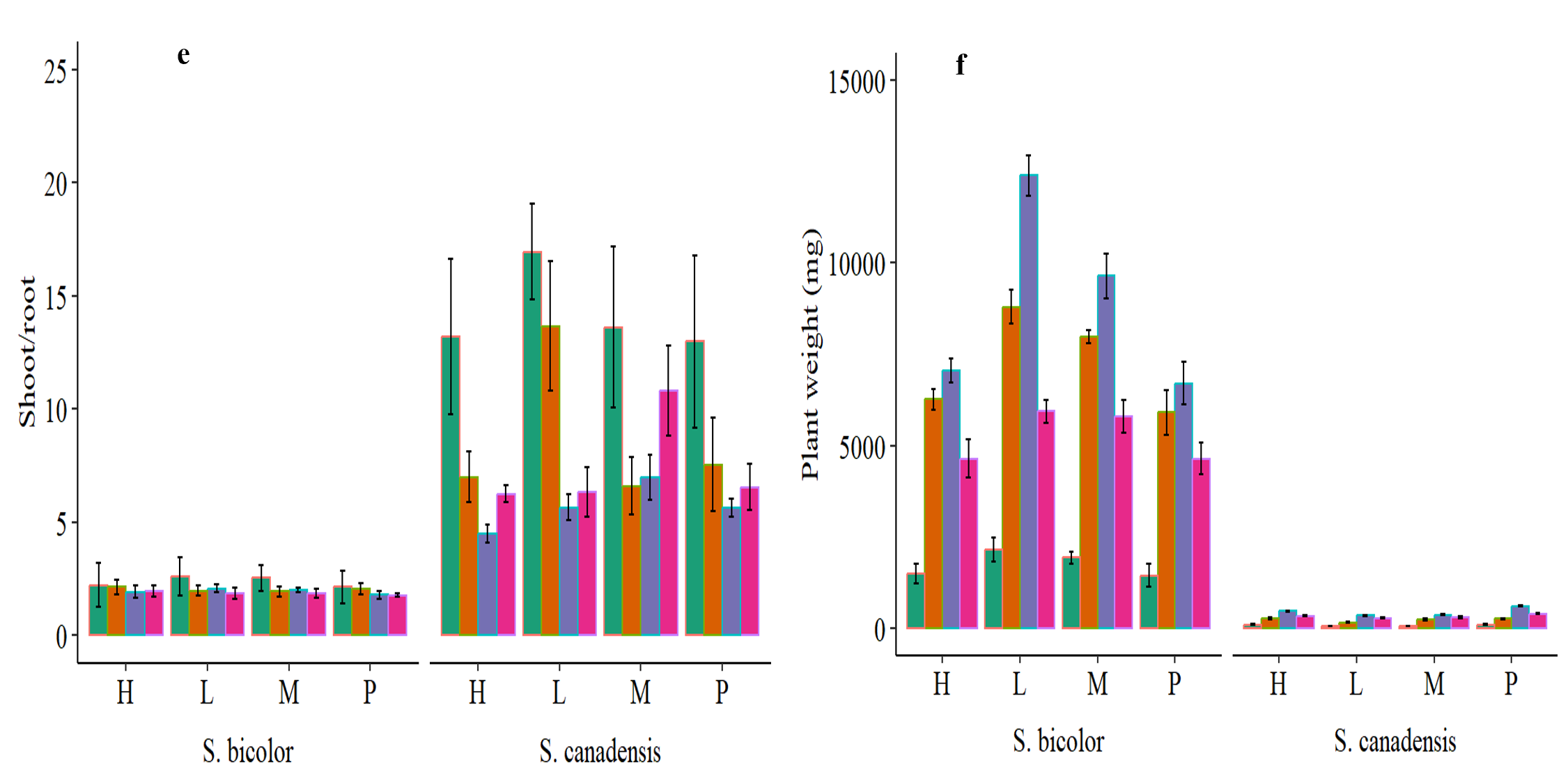


**Figure 6S:** Relative leaf chlorophyll content (a), transpiration rate (b), stomatal conductance (c), intracellular CO_2_ (d) , leaf length (e) and leaf width (f) of *Solidago canadensis* and *Sorghum bicolor* under varied invasion levels and available nitrogen forms. red colour: control; green colour: ammonical N; blue colour: both nitrogen form; purple colour: nitrate N; H: high invasion level; L: low invasion level; M: medium invasion level; P: no invasion.
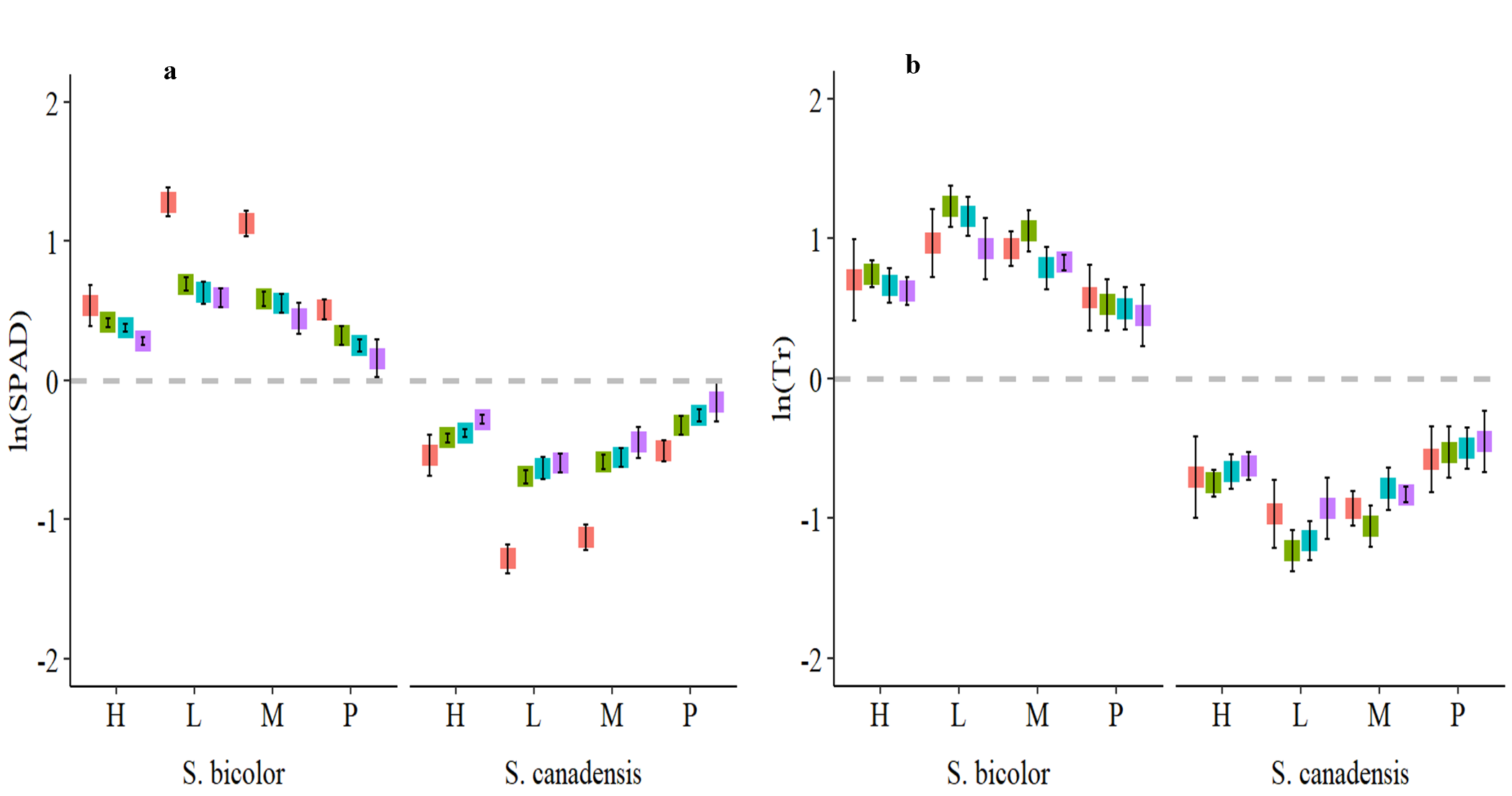

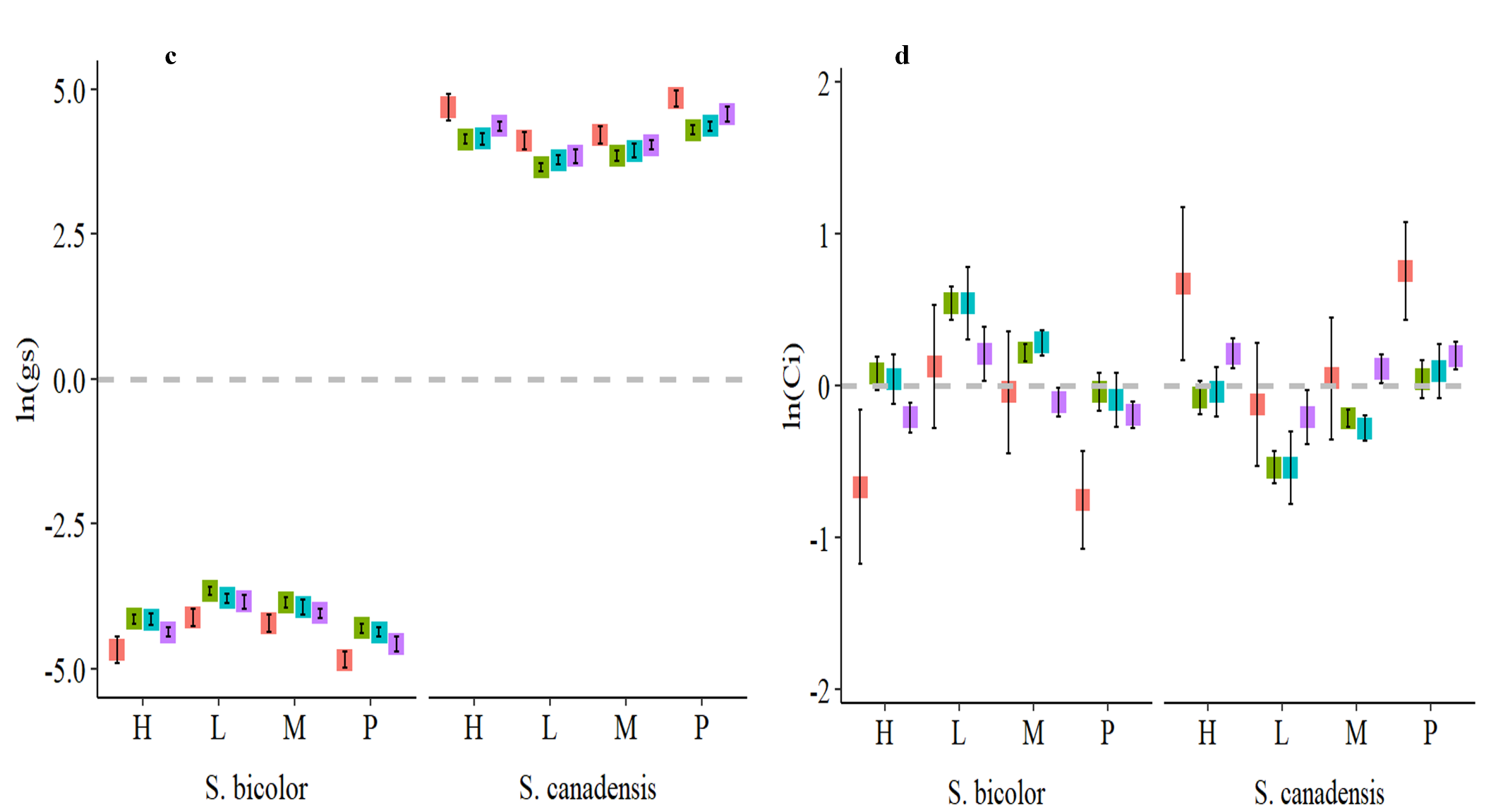

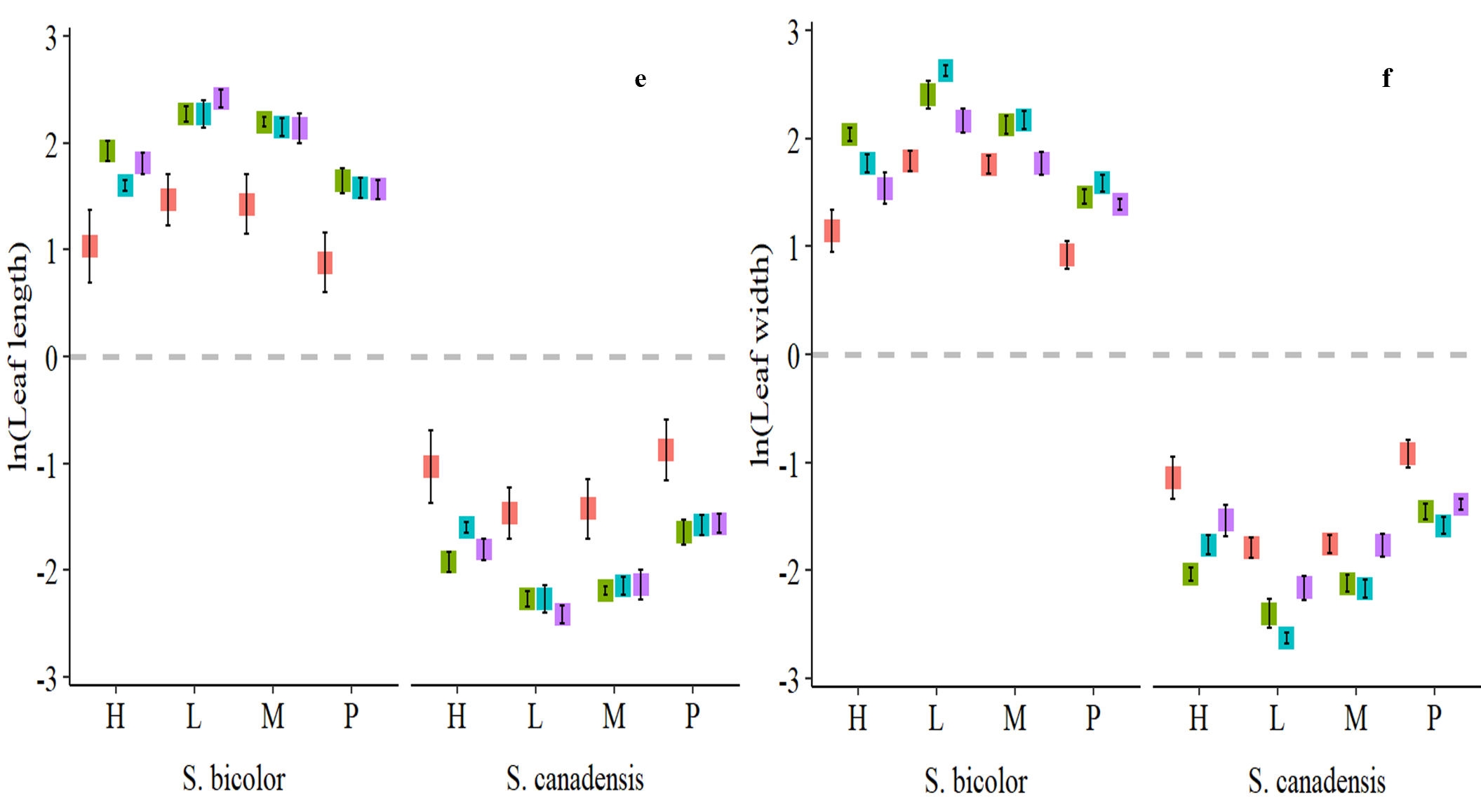


**Supplementary Table**

**Table S1:** The composition of modified Hogland solutions with different nitrogen forms supplied to *Solidago canadensis* and *Sorghum bicolor* under varied invasion levels.

| **Nutrients** | **Control**  **(g/L)** | **Nitrogen NO_3_ (g/L)** | **Nitrogen NH_4_ (g/L)** | **Nitrogen NH_4_+NO_3_ (g/L)** |
| --- | --- | --- | --- | --- |
| **CaNO_3_.4H_2_O** | - | 94.5 | - | 47.25 |
| **KNO_3_** | - | 50.6 | - | 25.3 |
| **NH_4_SO_4_** | - | - | 85.94 | 42.97 |
| **K2HPO4** | 13.6 | 13.6 | 13.6 | 13.6 |
| **MgSO4.7H2O** | 49.39 | 49.39 | 49.39 | 49.39 |
| **EDTA-FeNa.H2O** | 8.42 | 8.42 | 8.42 | 8.42 |
| **KI** | 0.00083 | 0.00083 | 0.00083 | 0.00083 |
| **MnSO4** | 0.0223 | 0.0223 | 0.0223 | 0.0223 |
| **Na2MoO4** | 0.00025 | 0.00025 | 0.00025 | 0.00025 |
| **CoO** | 0.00025 | 0.00025 | 0.00025 | 0.00025 |
| **H_3_BO_3_** | 0.0062 | 0.0062 | 0.0062 | 0.0062 |
| **ZnSO4** | 0.0086 | 0.0086 | 0.0086 | 0.0086 |
| **CuSO4** | 0.00025 | 0.00025 | 0.00025 | 0.00025 |

**Table S2:** Leaf chlorophyll contents, transpiration rate, stomatal conductance, intracellular CO_2_, leaf length and leaf width of *Solidago canadensis* and *Sorghum bicolor* under varied invasion levels and available nitrogen forms for plant species (P) invasion levels (I), nitrogen forms (F).

| **SOV** | **Df** | **SPAD** | **Tr** | **gs** | **Ci** | **Leaf length** | **Leaf width** |
| --- | --- | --- | --- | --- | --- | --- | --- |
| **P** | 1 | 57.53** | 120.82** | 3354.05** | 0.01ns | 602.94** | 614.99** |
| **I** | 3 | 0ns | 0ns | 0ns | 0ns | 0ns | 0ns |
| **F** | 3 | 0ns | 0ns | 0ns | 0ns | 0ns | 0ns |
| **P*I** | 3 | 2.53** | 2.88** | 4.43** | 3.86** | 4.96** | 7.47** |
| **P*F** | 3 | 2.29** | 0.27** | 2.14** | 3.12** | 7.05** | 4.21** |
| **I*F** | 9 | 0ns | 0ns | 0ns | 0ns | 0ns | 0ns |
| **P*I*F** | 9 | 0.17** | 0.07* | 0.03* | 0.2** | 0.08** | 0.21** |
| **Residual** | 160 | 0.01 | 0.03 | 0.01 | 0.06 | 0.03 | 0.01 |

**Note:** Mean square values of SPAD, Tr, gs, Ci, leaf length and leaf width; *, ** and ns represented the significance level at p<0.01, p<0.05 and non-significant (p>0.05) respectively. SOV: source of variations; Df: degree of freedom; Tr: transpiration rate; gs: stomatal conductance: Ci: intracellular CO_2_
